# Alcohol Consumption and Cardiovascular Disease: A Mendelian Randomization Study

**DOI:** 10.1161/CIRCGEN.119.002814

**Published:** 2020-05-05

**Authors:** Susanna C. Larsson, Stephen Burgess, Amy M. Mason, Karl Michaëlsson

**Affiliations:** 1 Department of Surgical Sciences, Uppsala University, Uppsala, Sweden (S.C.L., K.M.).; 2 Unit of Cardiovascular and Nutritional Epidemiology, Institute of Environmental Medicine, Karolinska Institutet, Stockholm, Sweden (S.C.L.).; 3 Department of Public Health and Primary Care, and MRC Biostatistics Unit, University of Cambridge, Cambridge, United Kingdom (S.B.).; 4 British Heart Foundation Cardiovascular Epidemiology Unit, Department of Public Health and Primary Care, University of Cambridge, Cambridge, United Kingdom (A.M.M.).; 5 National Institute for Health Research Cambridge Biomedical Research Centre, University of Cambridge and Cambridge University Hospitals, Cambridge, United Kingdom (A.M.M.).

**Keywords:** alcohol drinking, cardiovascular diseases, coronary artery disease, odds ratio, peripheral artery disease, stroke

## Abstract

The causal role of alcohol consumption for cardiovascular disease remains unclear. We used Mendelian randomization (MR) to predict the effect of alcohol consumption on 8 cardiovascular diseases.

**Methods:**

Up to 94 single-nucleotide polymorphisms were used as instrumental variables for alcohol consumption. Genetic association estimates for cardiovascular diseases were obtained from large-scale consortia and UK Biobank. Analyses were conducted using the inverse variance–weighted, weighted median, MR-PRESSO, MR-Egger, and multivariable MR methods.

**Results:**

Genetically predicted alcohol consumption was consistently associated with stroke and peripheral artery disease across the different analyses. The odds ratios (ORs) per 1-SD increase of log-transformed alcoholic drinks per week were 1.27 ([95% CI, 1.12–1.45] *P*=2.87×10^−4^) for stroke and 3.05 ([95% CI, 1.92–4.85] *P*=2.30×10^−6^) for peripheral artery disease in the inverse variance–weighted analysis. There was some evidence for positive associations of genetically predicted alcohol consumption with coronary artery disease (OR, 1.16 [95% CI, 1.00–1.36]; *P*=0.052), atrial fibrillation (OR, 1.17 [95% CI, 1.00–1.37]; *P*=0.050), and abdominal aortic aneurysm (OR, 2.60 [95% CI, 1.15–5.89]; *P*=0.022) in the inverse variance–weighted analysis. These associations were somewhat attenuated in multivariable MR analysis adjusted for smoking initiation. There was no evidence of associations of genetically predicted alcohol consumption with heart failure (OR, 1.00 [95% CI, 0.68–1.47]; *P*=0.996), venous thromboembolism (OR, 1.04 [95% CI, 0.77–1.39]; *P*=0.810), and aortic valve stenosis (OR, 1.03 [95% CI, 0.56–1.90]; *P*=0.926).

**Conclusions:**

This study provides evidence of a causal relationship between higher alcohol consumption and increased risk of stroke and peripheral artery disease. The causal role of alcohol consumption for other cardiovascular diseases requires further research.

## Abstract 2

Supplemental Digital Content is available in the text.

Heavy alcohol consumption is an important cause of death and disability,^1^ but the association between moderate drinking and cardiovascular disease (CVD) is complex. On a population level, given its widespread nature, it is important to disentangle any risks or benefits of alcohol consumption. Observational studies have generally shown that alcohol consumption is positively associated with risk of atrial fibrillation,^2^ heart failure,^3^ and hemorrhagic stroke,^4^ whereas moderate drinking is associated with lower risk of coronary heart disease and ischemic stroke.^3–6^ Data from observational studies on alcohol consumption in relation to other CVDs, including venous thromboembolism,^7,8^ peripheral artery disease,^9^ aortic valve stenosis,^10^ and abdominal aortic aneurysm,^9,11,12^ are limited or inconsistent. Observational studies are unable to fully account for confounding and reverse causation bias, and, therefore, causality in the associations of alcohol consumption with different CVDs remains uncertain. Furthermore, self-reported alcohol consumption may be underestimated, leading to measurement error in the assessment of alcohol consumption, which may result in attenuated categorical risk estimates.

Mendelian randomization (MR) is an epidemiological technique that utilizes genetic variants that are reliably associated with a potentially modifiable risk factor to determine its causal role for disease risk.^13^ MR studies are less vulnerable to bias from confounding, reverse causation, and measurement error compared with conventional observational studies. In a previous MR study in a population of European ancestry, increased alcohol intake instrumented by a single-nucleotide polymorphism (SNP; rs1229984) in the *ADH1B* gene was associated with higher risk of coronary heart disease and ischemic stroke,^14^ which contradicts observational findings.^3–6^ Results from a recent MR study conducted in a Chinese population showed that increased alcohol consumption instrumented by 2 alcohol-associated SNPs (rs1229984 in *ADH1B* and rs671 in *ALDH2*) was associated with higher risk of ischemic stroke and intracerebral hemorrhage but was not associated with myocardial infarction.^15^ To our knowledge, the association between alcohol consumption and CVD other than stroke and coronary heart disease has not been studied using MR.

A recent genome-wide association meta-analysis identified multiple SNPs associated with alcohol consumption.^16^ By using those SNPs as instrumental variables, we here perform an MR study to investigate the potential causal relationship between alcohol consumption and 8 CVDs, including stroke, coronary artery disease, atrial fibrillation, heart failure, venous thromboembolism, peripheral artery disease, aortic valve stenosis, and abdominal aortic aneurysm. In secondary analyses, we explored the associations of genetically predicted alcohol consumption with possible mediators (blood pressure and serum lipids) and confounders (smoking and education) of the alcohol-CVD associations.

## Methods

**Figure 1.**
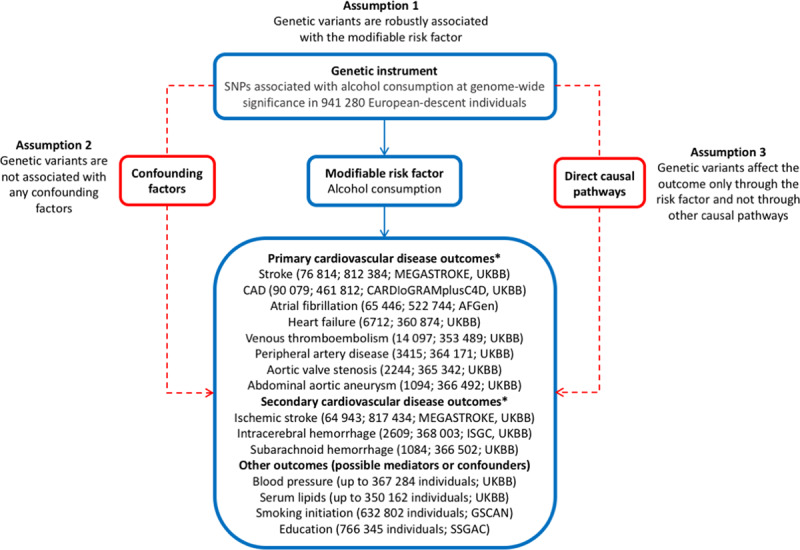
**Summary of the data sources for this study and the assumptions for the Mendelian randomization design.** Broken lines represent potential pleiotropic or direct causal effects between variables that would violate the Mendelian randomization assumptions. AFGen indicates Atrial Fibrillation Consortium; CAD, coronary artery disease; CARDIoGRAMplusC4D, Coronary Artery Disease Genome-Wide Replication and Meta-Analysis Plus the Coronary Artery Disease Genetics Consortium; GSCAN, GWAS and Sequencing Consortium of Alcohol and Nicotine Use; GWAS, genome-wide association study; ISGC, International Stroke Genetics Consortium; SNP, single-nucleotide polymorphism; SSGAC, Science Genetic Association Consortium; and UKBB, UK Biobank. *Reported are the outcome and within parenthesis, the number of cases and noncases and data source(s).

The methods used in this MR study are described in the Data Supplement. In brief, summary statistics data for alcohol consumption and the outcomes were obtained from meta-analyses of genome-wide association studies and UK Biobank. All studies included in the genome-wide association studies were approved by an institutional review committee, and participants gave informed consent. This MR study was approved by the Swedish Ethical Review Authority. The data that support the findings of this study are available from the corresponding author upon reasonable request. Figure 1 provides an overview of this study, including the primary and secondary outcomes studied, the data sources used, and the principles and assumptions of the MR design.

## Results

### Associations With Potential Mediators and Confounders

**Figure 2.**
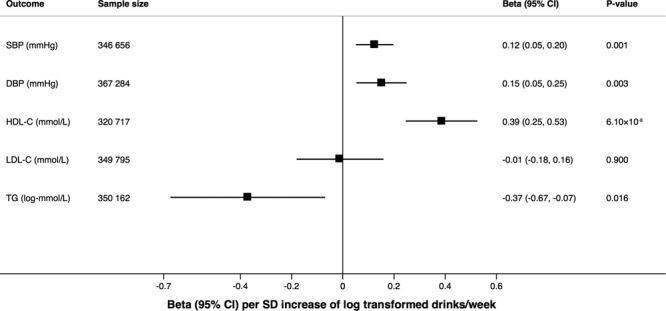
**Associations of alcohol consumption instrumented by the full set of single-nucleotide polymorphisms with blood pressure and lipids.** The effect estimates are per SD increase of log-transformed alcoholic drinks per week, and results are based on the random-effects inverse variance–weighted method. DBP indicates diastolic blood pressure; HDL-C, high-density lipoprotein cholesterol; LDL-C, low-density lipoprotein cholesterol; OR, odds ratio; SBP, systolic blood pressure; and TG, triglyceride.

Alcohol consumption instrumented by the full set of SNPs (n=94) was positively associated with systolic and diastolic blood pressure and high-density lipoprotein cholesterol levels, inversely associated with triglyceride levels, but not associated with low-density lipoprotein cholesterol levels (Figure 2; Table VIII in the Data Supplement). There was strong evidence of association of the overall genetic instrument for alcohol consumption with smoking initiation (odds ratio [OR], 1.24 [95% CI, 1.15–1.33]; *P*=9.5×10^−9^) and weak evidence for education (β=−0.09 years of schooling completed [95% CI, −0.17 to 0.00]; *P*=0.05).

Alcohol consumption instrumented by rs1229984 in *ADH1B* was positively associated with systolic and diastolic blood pressure and low-density lipoprotein cholesterol levels but was not associated with high-density lipoprotein cholesterol or triglyceride levels (Table IX in the Data Supplement). Genetically predicted alcohol consumption based on the *ADH1B* variant was not associated with smoking initiation (OR, 0.97 [95% CI, 0.89–1.07]; *P*=0.57) but was inversely associated with education (β=−0.15 years of schooling completed [95% CI, −0.23 to −0.08]; *P*=5.4×10^−5^).

### Associations With CVD

**Figure 3.**
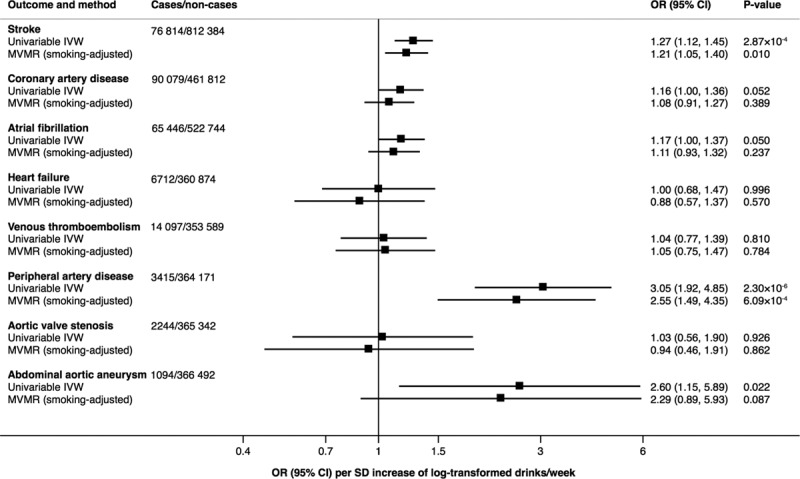
**Associations of alcohol consumption instrumented by the full set of single-nucleotide polymorphisms with CVD.** Odds ratios (ORs) are per SD increase of log-transformed alcoholic drinks per week, and results are based on the random-effects inverse variance–weighted (IVW) method and multivariable Mendelian randomization (MVMR) analysis adjusted for smoking initiation.

Alcohol consumption instrumented by the full set of SNPs was consistently associated with stroke and peripheral artery disease across different analyses (Figure 3; Table X in the Data Supplement). The ORs per 1-SD increase in log-transformed drinks per week were 1.27 ([95% CI, 1.12–1.45] *P*=2.87×10^−4^) for stroke and 3.05 ([95% CI, 1.92–4.85] *P*=2.30×10^−6^) for peripheral artery disease in the univariable inverse variance–weighted analysis. There was some evidence for positive associations of genetically predicted alcohol consumption with coronary artery disease (OR, 1.16 [95% CI, 1.00–1.36]; *P*=0.052), atrial fibrillation (OR, 1.17 [95% CI, 1.00–1.37]; *P*=0.050), and abdominal aortic aneurysm (OR, 2.60 [95% CI, 1.15–5.89]; *P*=0.022) in the univariable inverse variance–weighted analysis (Figure 3; Table X in the Data Supplement). These associations were somewhat attenuated in multivariable MR analysis adjusting for smoking initiation (Figure 3; Table X in the Data Supplement). There was no evidence of association of genetically predicted alcohol consumption with heart failure (OR, 1.00 [95% CI, 0.68–1.47]; *P*=0.996), venous thromboembolism (OR, 1.04 [95% CI, 0.77–1.39]; *P*=0.810), and aortic valve stenosis (OR, 1.03 [95% CI, 0.56–1.90]; *P*=0.926) in the inverse variance–weighted analysis (Figure 3), but suggestive positive associations with aortic valve stenosis were observed in the weighted median and MR-Egger analyses (Table X in the Data Supplement). Indication of directional pleiotropy was only detected in the analysis of aortic valve stenosis (Table X in the Data Supplement). The associations between genetically predicted alcohol consumption and stroke and coronary artery disease were similar when using data from the consortia (MEGASTROKE and Coronary Artery Disease Genome-Wide Replication and Meta-Analysis Plus the Coronary Artery Disease Genetics Consortium [CARDIoGRAMplusC4D]) and UK Biobank (Figure I in the Data Supplement). Restricting the analyses to never smokers in UK Biobank (Table XI in the Data Supplement) yielded ORs that were similar to the smoking-adjusted estimates, except for the association with coronary artery disease, which became stronger (OR, 1.39 [95% CI, 0.98–1.96]; *P*=0.064).

**Figure 4.**
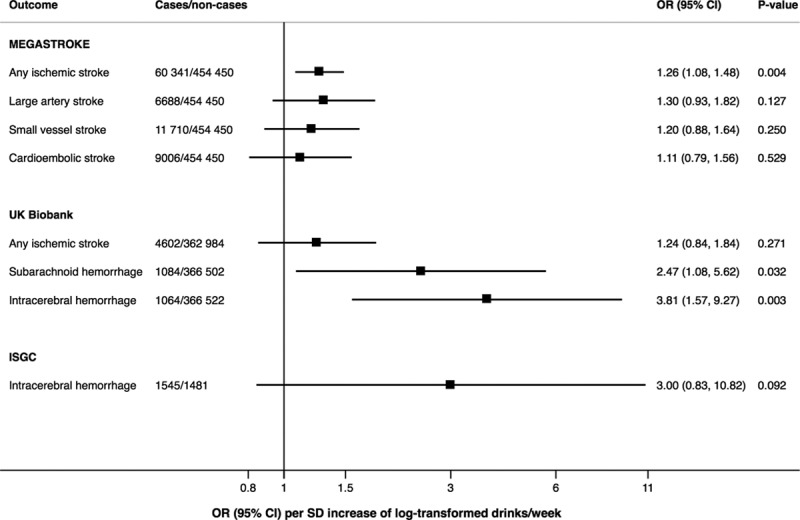
**Associations of alcohol consumption instrumented by the full set of single-nucleotide polymorphisms with stroke types in the MEGASTROKE Consortium, UK Biobank, and International Stroke Genetics Consortium (ISGC).** Odds ratios (ORs) are per SD increase of log-transformed alcoholic drinks per week, and results are based on the random-effects inverse variance–weighted method.

The associations of alcohol consumption instrumented by all SNPs with stroke types in the MEGASTROKE Consortium, UK Biobank, and International Stroke Genetics Consortium are presented in Figure 4. The OR for ischemic stroke combining the results from MEGASTROKE and UK Biobank was 1.26 ([95% CI, 1.09–1.46] *P*=0.002). The corresponding OR for intracerebral hemorrhage, combining the results from the International Stroke Genetics Consortium and UK Biobank, was 3.53 ([95% CI, 1.70–7.32] *P*=0.001). Results for stroke and atrial fibrillation based on data from the MEGASTROKE Consortium and Atrial Fibrillation Consortium, respectively, were similar when restricting the study samples to European-descent individuals (Table XII in the Data Supplement).

Alcohol consumption instrumented by rs1229984 in *ADH1B* was strongly or suggestively associated with higher odds of any stroke, coronary artery disease, peripheral artery disease, aortic valve stenosis, and abdominal aortic aneurysm (all *P*<0.05; Table XIII in the Data Supplement). There was suggestive evidence of a positive association of alcohol consumption instrumented by rs1229984 with ischemic stroke (OR, 1.31 [95% CI, 1.05–1.65]; *P*=0.018) and intracerebral hemorrhage (OR, 11.5 [95% CI, 1.31–101.46]; *P*=0.027). Results for the associations of the overall genetic instrument for alcohol consumption but with exclusion of rs1229984 with CVD were similar (Table XIV in the Data Supplement) to those based on the full set of SNPs.

## Discussion

### Principal Findings

This MR study provides evidence that higher alcohol consumption may be causally associated with increased risk of stroke and peripheral artery disease. There was also suggestive evidence for positive associations of genetically predicted alcohol consumption with coronary artery disease, atrial fibrillation, and abdominal aortic aneurysm, but the associations were attenuated after adjustment for smoking. Alcohol consumption instrumented by the full set of variants was additionally associated with higher blood pressure and high-density lipoprotein cholesterol levels and with lower triglyceride levels.

### Comparison With Other Studies

Our findings for alcohol consumption, based on the *ADH1B* gene variant, in relation to stroke and coronary artery disease largely confirm the results from 2 earlier MR studies that utilized 1 or 2 SNPs as instrumental variables for alcohol consumption.^14,15^ An MR analysis of 261 991 European-descent individuals, including 20 259 coronary heart disease cases, found that each additional alcohol-decreasing allele of rs1229984 in the *ADH1B* gene was associated with a 10% lower odds of coronary heart disease.^14^ In the larger dataset (with 3× as many cases of coronary artery disease) used in the present MR analysis, we also observed that alcohol consumption instrumented by rs1229984 was associated with coronary artery disease. In a cohort study of 161 490 Chinese adults (China Kadoorie Biobank) genotyped for 2 alcohol-associated SNPs (rs1229984 in *ADH1B* and rs671 in *ALDH2*), genetically predicted higher alcohol consumption was not associated with myocardial infarction but was associated with higher risk of stroke, particularly intracerebral hemorrhage.^15^ The present study confirms that those findings for stroke are also valid for individuals of European ancestry and that the association is stronger for intracerebral hemorrhage than ischemic stroke. The frequency of the alcohol-decreasing allele of rs1229984 is considerably higher in East Asians (overall frequency of 0.69 in China Kadoorie Biobank)^15^ than in individuals of European ancestry (0.026 in UK Biobank). The alcohol-decreasing allele of rs671 in the *ALDH2* gene is not found in European populations. We are not aware of any previous MR analysis of alcohol consumption in relation to risk of atrial fibrillation, heart failure, venous thromboembolism, peripheral artery disease, aortic valve stenosis, or abdominal aortic aneurysm.

The reason why rs1229984 in *ADH1B* was strongly associated with coronary artery disease, whereas only a suggestive association was observed between the overall genetic instrument for alcohol consumption and coronary artery disease, is unclear. However, the alcohol-increasing allele of rs1229984 was inversely associated with education, which could potentially explain the associations through horizontal pleiotropy. Rs1229984 was also associated with low-density lipoprotein cholesterol, and both this variant and the overall genetic instrument for alcohol consumption were associated with blood pressure. However, these associations may represent vertical (mediated) pleiotropy.

### Possible Mechanisms

A possible mechanism whereby alcohol consumption may increase the risk of CVD is through blood pressure. A meta-analysis of randomized trials showed that a reduction in alcohol intake decreased blood pressure in a dose-response manner in individuals who drank >2 alcoholic drinks per day.^17^ In addition, genetically predicted alcohol consumption was positively associated with systolic blood pressure in previous MR studies,^14,15^ and results were replicated by our study with both the overall genetic instrument for alcohol consumption and the *ADH1B* gene variant.

High-impact binge alcohol drinking is associated with increased odds of clinically high total serum cholesterol and triglyceride levels.^18^ In contrast, short-term interventional studies have shown that moderate alcohol drinking leads to favorable changes in several cardiovascular biomarkers, including higher levels of high-density lipoprotein cholesterol and adiponectin and lower fibrinogen levels.^19^ We confirmed a positive association between genetically predicted alcohol intake and higher levels of high-density lipoprotein cholesterol. We additionally found that genetically higher alcohol consumption was associated with lower triglyceride levels. A causal positive association between triglyceride levels and risk of coronary artery disease^20^ but not ischemic stroke^21^ has been shown in MR studies.

### Strengths and Limitations

Strengths of this study include the use of data from large studies of alcohol consumption and the outcomes, the MR study design, and the use of multiple SNPs as instrumental variables for alcohol consumption. The MR approach reduces bias due to reverse causality and confounding. Reverse causality is minimized in MR studies because disease cannot modify genotype, which is fixed at conception. With regard to confounding factors, self-reported alcohol consumption is associated with higher educational attainment.^6,22^ In contrast, there is no genetic correlation between education and alcohol consumption (*r*_g_=0.01),^16^ and we found no association of the overall genetic instrument for alcohol consumption with education in this MR study. However, the *ADH1B* gene variant was associated with education. The overall genetic instrument for alcohol consumption was associated with smoking initiation, and adjustment for genetic predisposition to smoking attenuated the estimates for alcohol consumption and most CVDs. If the association between genetically predicted alcohol consumption and smoking partly or entirely reflects vertical (mediated) pleiotropy rather than horizontal pleiotropy, adjustment for smoking may have attenuated the full effect of alcohol consumption on risk of CVD. The smoking-adjusted estimates can be interpreted as the direct effect of alcohol consumption on CVD not mediated by smoking.

The validity of the results of an MR study depends on the assumptions that the instrumental variables are robustly associated with the risk factor and that pleiotropic or other direct causal pathways do not explain the association with the outcome (Figure 1). In the present study, we only used SNPs that were associated with alcohol consumption at a genome-wide significance level.^16^ Furthermore, the associations of genetically predicted alcohol consumption with stroke and peripheral artery disease were similar in different MR analyses and remained after adjusting for smoking and in never smokers, suggesting that false positive findings due to horizontal pleiotropy via smoking are unlikely.

A limitation of this MR study is that despite large sample sizes, the precision was low in the analyses of outcomes with few cases. Another shortcoming is that UK Biobank participants were included in the dataset in which the genetic variants were derived and in both the exposure and outcome datasets for most analyses. This might have introduced bias in the causal estimates. However, genetic associations with outcomes in independent datasets should not be affected by this sample overlap. Results for stroke and coronary artery disease were similar in analyses confined to data from the MEGASTROKE and CARDIoGRAMplusC4D consortia. Results were also similar using only the rs1229984 variant, which was not discovered in UK Biobank. A further limitation is that we cannot rule out that population stratification may have had some effect on the results. However, the vast majority of participants were of European ancestry, and the results were similar in analyses restricted to European-descent individuals. Finally, we could not investigate potential U-shaped or J-shaped associations. This precludes us from making specific quantitative statements about the relative harm of moderate drinking versus heavy drinking. However, as the prevalence of heavy drinking in the UK Biobank was low, it is unlikely that the burden of increased disease risk is restricted to heavy drinkers.

### Conclusions

This study provides evidence of a causal relationship between higher alcohol consumption and increased risk of stroke and peripheral artery disease. The causal role of alcohol consumption for other CVDs requires further research.

## Acknowledgments

The analyses of UK Biobank data were conducted under application 29202. Summary statistics data for genetic associations with stroke, coronary artery disease, and atrial fibrillation have been contributed by the MEGASTROKE Consortium, the Coronary Artery Disease Genome-Wide Replication and Meta-Analysis plus the Coronary Artery Disease Genetics Consortium, and the Atrial Fibrillation Consortium. The investigator list of the MEGASTROKE Consortium is available at http://megastroke.org/authors.html. Funding sources of the MEGASTROKE project are specified at megastroke.org/acknowledgements.html.

## Sources of Funding

This study was supported by research grants from the Swedish Research Council (Vetenskapsrådet; grant No. 2019-00977), the Swedish Research Council for Health, Working Life and Welfare (Forte; grant No. 2018-00123), and the Swedish Heart-Lung Foundation (Hjärt-Lungfonden; grant No. 20190247). Dr Burgess is supported by a Sir Henry Dale Fellowship jointly funded by the Wellcome Trust and the Royal Society (grant No. 204623/Z/16/Z).

## Disclosures

None.

## Supplementary Material


