# Supplementary material for: Alcohol Consumption and Cardiovascular Disease: A Mendelian Randomization Study
Source: Circ Genom Precis Med. 2020 May 5;13(3):e002814. doi: 10.1161/CIRCGEN.119.002814 (PMC7299220; doi:10.1161/CIRCGEN.119.002814)
Supplement: Supplementary file 1 [file hcg-13-e002814-s001.pdf]

SUPPLEMENTAL MATERIAL

METHODS

Data Sources

Detailed information on the data sources used in this Mendelian randomization (MR) study is available in Table I and Table II. In brief, summary statistics data for alcohol consumption, smoking initiation, and education were available from meta-analyses of genome-wide association studies conducted by the GWAS and Sequencing Consortium of Alcohol and Nicotine use (GSCAN)[1] and the Social Science Genetic Association Consortium (SSGAC).[2] The corresponding data for genetic associations with the cardiovascular diseases (CVDs) were obtained from the UK Biobank[3] and the following consortia: the MEGASTROKE Consortium with data on any stroke and ischemic stroke and its subtypes;[4] International Stroke Genetics Consortium (ISGC) with data on intracerebral hemorrhage;[5] the Coronary ARtery DIsease Genome-wide Replication and Meta-analysis plus The Coronary Artery Disease Genetics (CARDIoGRAMplusC4D) consortium with data on coronary artery disease;[6] and the Atrial Fibrillation Consortium (AFGen) with data on atrial fibrillation.[7] The datasets included mainly or only European-descent individuals, and most genetic association analyses were adjusted for principal components of ancestry and additionally for age and sex in some studies (Table I). From UK Biobank, we used data for systolic and diastolic pressure, serum lipids (high-density lipoprotein cholesterol, low-density lipoprotein cholesterol, and triglycerides), and all primary CVD outcomes except atrial fibrillation because participants of UK Biobank were included in the AFGen. UK Biobank participants were also included in the GSCAN and SSGAC but not in the MEGASTROKE Consortium or the CARDIoGRAMplusC4D consortium.

Selection of Instrumental Variables

The GSCAN genome-wide association study identified 99 conditionally independent single-nucleotide polymorphisms (SNPs) associated with alcohol consumption (log-transformed alcoholic drinks per week) at the genome-wide significance threshold ($P<5\times10^{-8}$) in 941 280 individuals of European ancestry.[1] Linkage disequilibrium (defined as $r^2>0.1$) between the SNPs was assessed using LDlink[8] and was found among four SNP pairs. The SNP within each correlated pair that had the weakest association with alcohol consumption was excluded, leaving 95 independent SNPs for further consideration. Up to five SNPs were unavailable in the primary CVD outcome datasets. A suitable proxy SNP ($r^2>0.95$ with the specified SNP) was available for two SNPs. Hence, summarized data for 92 to 94 SNPs were used in the main analyses. Eighty-one SNPs were available for analyses of intracerebral hemorrhage in the International Stroke Genetics Consortium. The SNPs used as instrumental variables and their associations with the outcomes are shown in Tables III to VII.

Statistical Analysis

Five complementary MR methods with distinct strengths and assumptions were used for the analyses of genetically-predicted alcohol consumption in relation to the eight primary CVD outcomes: (1) the inverse-variance weighted method (under a multiplicative random-effects model), which provides the highest precision but assumes that all SNPs are valid instrumental

variables[9]; (2) the weighted median method, which provides less precise estimates than the inverse-variance weighted method but gives reliable estimates if at least half of the weight in the analysis comes from valid instrumental variables[9]; (3) the MR-Egger method, which has low precision but can detect and correct for pleiotropy and provide a causal estimate even if all genetic variants have pleiotropic effects on the outcome[9]; (4) the MR-PRESSO (Pleiotropy RESidual Sum and Outlier) method, which can identify and correct for horizontal pleiotropy through removal of outliers;[10] and (5) a multivariable MR approach[11] to adjust for smoking initiation, which is genetically correlated with alcohol consumption (r_g=0.36).[1] In multivariable MR, the outcome is regressed on genetically-predicted values of alcohol consumption and smoking initiation, meaning that estimates represent the direct effect of alcohol consumption that is not mediated via smoking initiation.[12] Summary statistics data for smoking initiation were obtained from the GSCAN.[1] We further conducted a sensitivity analysis by calculating genetic associations with CVDs in never smokers in UK Biobank. In the primary analyses of the consortia data for any stroke,[4] ischemic stroke,[4] and atrial fibrillation,[7] we used the multi-ancestry datasets to increase power. In sensitivity analyses, the European-only datasets were used. European-only data were not available from the CARDIoGRAMplusC4D consortium.[6] The dataset for intracerebral hemorrhage included individuals of European ancestry only.[5] The analyses were carried out using the mrrobust,[13] MRPRESSO[10] and MendelianRandomization[14] packages. In a further secondary analysis, rs1229984 (the SNP with the strongest association with alcohol intake) in the *ADH1B* gene was used as an instrumental variable for alcohol consumption. Furthermore, the analyses for the overall genetic instrument were repeated after exclusion of rs1229984. All reported results for alcohol consumption are scaled to a standard deviation increase of log-transformed drinks per week. Results for stroke and coronary artery disease based on the MEGASTROKE and CARDIoGRAMplusC4D consortia datasets were combined with the corresponding results from UK Biobank by using fixed-effects inverse-variance weighted meta-analysis. All statistical tests were two-sided. Bonferroni-corrected *P* values below 0.006 (where α=0.05/8 CVD outcomes) were considered strong evidence of associations and *P* values between 0.006 and 0.05 as suggestive evidence of associations.

Power Calculation and Instrumental Strength

Statistical power was estimated by using a web-based application.[15] The power calculation, assuming a variance of 0.25% explained by the alcohol-associated SNPs[1], showed that we had 80% power (at α=0.05) to detect an OR of 1.22 for any stroke, 1.21 for coronary artery disease, 1.24 for atrial fibrillation, 1.70 for heart failure, 1.50 for venous thromboembolism, 1.95 for peripheral artery disease, 2.20 for aortic valve stenosis and 2.70 for abdominal aortic aneurysm. The F-statistic estimated in UK Biobank was 29, indicating that the genetic instrument was sufficient strong to mitigate weak instrument bias.

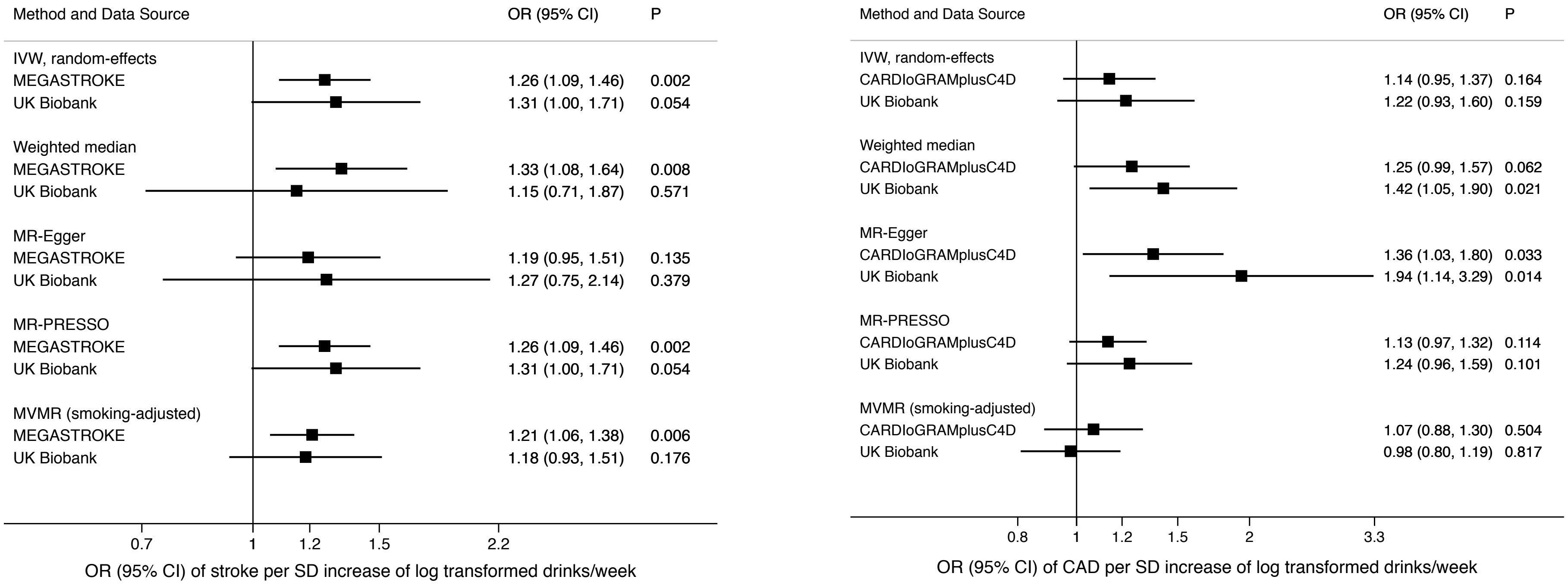

Figure I. Associations of alcohol consumption instrumented by the full set of single-nucleotide polymorphisms with stroke (left figure) and coronary artery disease (right figure) in Mendelian randomization analyses based on data from the MEGASTROKE and CARDIoGRAMplusC4D consortia and UK Biobank. Odds ratios are per one standard deviation increment of log transformed alcoholic drinks per week. CI, confidence interval; IVW, inverse-variance weighted; MR-PRESSO, Mendelian randomization Pleiotropy RESidual Sum and Outlier; MVMR, multivariable Mendelian randomization; OR, odds ratio.

Table I. Details of the datasets used in the present Mendelian randomization study

Exposure/Outcome(s)	Consortium or cohort	Participants	Exposure/Outcome definition	Adjustments	Web source
Alcohol consumption (the exposure in this Mendelian randomization study)	GWAS and Sequencing Consortium of Alcohol and Nicotine use (GSCAN)	941 280 European-descent individuals	Average number of drinks consumed each week. Log-transformed prior to analysis	Age, age squared, sex and genetic principal components	https://conservancy.umn.edu/handle/11299/201564
Smoking	GWAS and Sequencing Consortium of Alcohol and Nicotine use (GSCAN)	632 802 European-descent individuals	Smoked over 100 cigarettes over the life course, ever smoked every day for at least a month, or ever smoked regularly	Age, age squared, sex and genetic principal components	https://conservancy.umn.edu/handle/11299/201564
Education	Social Science Genetic Association Consortium (SSGAC)	766 345 European-descent individuals	Number of years of schooling completed, constructed by imputing a years-of-education equivalent for each International Standard Classification of Education system category	Age, sex and genetic principal components	https://www.thessgac.org/data
Any stroke, ischemic stroke, and ischemic stroke subtypes	MEGASTROKE Consortium	521 612 individuals (67 162 stroke cases and 454 450 non-cases) of mainly European (86%) and Asian (9%) ancestry; 60 341 cases of any ischemic stroke, 6688 large artery stroke, 11 710 small vessel stroke and 9006 cardioembolic stroke	The classification of ischemic stroke subtypes was based on the Trial of Org 10172 in Acute Stroke Treatment (TOAST) criteria	Minimum of age and sex	http://www.megastroke.org

Intracerebral hemorrhage	International Stroke Genetics Consortium (ISGC)	1545 cases of intracerebral hemorrhage and 1481 controls of European ancestry	Spontaneous intracerebral hemorrhage was defined as a new and acute neurological deficit with compatible brain imaging showing the presence of intraparenchymal bleeding	Age, sex, and genetic principal components	http://cerebrovascularportal.org/informational/downloads
Coronary artery disease	Coronary ARtery DIsease Genome-wide Replication and Meta-analysis plus The Coronary Artery Disease Genetics (CARDIoGRAMplusC4D) consortium	184 305 individuals (60 801 cases of coronary artery disease and 123 504 non-cases) of mainly European (77%) and Asian (19%) ancestry	Myocardial infarction, acute coronary syndrome, chronic stable angina, or coronary stenosis >50%	Information not available	www.cardiogramplusc4d.org/
Atrial fibrillation	Atrial Fibrillation Consortium (AFGen)	588 190 individuals (65 446 atrial fibrillation cases and 522 744 non-cases) of mainly European (91%) and Asian (6%) ancestry	Paroxysmal or permanent atrial fibrillation or atrial flutter	Age, sex, genotyping array and genetic principal components	http://www.broadcvdi.org/home/portalHome
Any stroke, ischemic stroke, and ischemic stroke subtypes	MEGASTROKE Consortium	521 612 individuals (67 162 stroke cases and 454450 non-cases) of mainly European (86%) and Asian (9%) ancestry; 60 341 cases of any ischemic stroke, 6688 large artery stroke, 11 710 small vessel stroke and 9006 cardioembolic stroke	The classification of ischemic stroke subtypes was based on the Trial of Org 10172 in Acute Stroke Treatment (TOAST) criteria	Minimum of age and sex	http://www.megastroke.org

Stroke, coronary artery disease, heart failure, venous thromboembolism, peripheral artery disease, aortic valve stenosis, and abdominal aortic aneurysm	UK Biobank	367 586 unrelated European-descent individuals. The number of cases is reported in Supplementary Table II.	See Table II	The first ten genetic principal components	https://www.ukbiobank.ac.uk/
Systolic and diastolic blood pressure, high-density and low-density lipoprotein cholesterol, triglycerides	UK Biobank	367 586 unrelated European-descent individuals	Not applicable	The first ten genetic principal components	https://www.ukbiobank.ac.uk/

Table II. Definitions and sources of information for cardiovascular diseases in UK Biobank

Outcome	Cases	ICD-9 diagnosis	ICD-10 diagnosis	OPCS procedure	Self-report†
Any stroke	9652	430, 431, 434, 436	I60, I61, I63, I64		20002
Ischemic stroke	4602	434, 436	I63, I64		20002
Intracerebral hemorrhage	1064	431	I61		20002
Subarachnoid hemorrhage	1084	430	I60		20002
Coronary artery disease	29 278	410, 411, 412, 414.0, 414.8, 414.9	I21, I22, I23, I24, I25.1, I25.2, I25.5, I25.6, I25.8, I25.9	K40, K41, K42, K43, K44, K45, K46, K49, K50.1, K50.2, K50.4, K75	20002, 20004, 6150
Heart failure	6712	402.01, 402.11, 402.91, 404.01, 404.11, 404.91, 404.03, 404.13, 404.93, 428	I11.0, I13.0, I13.2, I50		20002
Venous thromboembolism	14 097	415.1, 451.1, 452, 453.0, 453.4, 453.9	I26, I80.1, I80.2, I81, I82.0	L90.2	20002, 6152
Peripheral artery disease	3415	443.8, 443.9	I73.8, I73.9		20002
Aortic valve stenosis	2244		I35.0, I35.2		20002
Abdominal aortic aneurysm	1094	441.3, 441.4	I71.3, I71.4	L19.4, L19.5	20002

ICD, International Classification of Disease; OPCS, Office of Population Censuses and Surveys Classification of Surgical Operations and Procedures. *Follow-up for incident cases was until March 31, 2017 and date of death was recorded until February 14, 2018. †Numbers refer to data codes used in UK Biobank: 6150/6152 = Health condition diagnosed by doctor (self-reported); 6177 = Medication for health condition; 20002 = Non-cancer illness code (self-reported from interview with nurse); 20004 = Surgical operation code (self-reported from interview with nurse)

Table III. Association between alcohol-related SNPs and any stroke in the MEGASTROKE Consortium*

					Association with alcohol			Association with stroke		
SNP	Chr	Gene	EA	NEA	Beta†	SE		Beta†	SE	P
rs10753661	1	Intergenic	G	A	0,009	0,002		0,016	0,009	0,081
rs12088813	1	Intron:PDE4B	A	C	0,009	0,002		0,008	0,009	0,351
rs28680958	1	Intron:ZBTB37	G	A	0,011	0,002		0,002	0,009	0,865
rs5024204	1	Intron:PTGER3	T	A	0,010	0,002		0,018	0,008	0,034
rs58107686	1	Intron:PHC2	C	A	0,010	0,002		0,012	0,009	0,169
rs705687	1	Intergenic	A	G	0,011	0,002		0,003	0,009	0,754
rs823114	1	Intergenic	A	G	0,009	0,002		0,008	0,008	0,328
rs11692435	2	Nonsynonymous:ACTR1B	A	G	0,017	0,003		0,000	0,017	0,988
rs1260326	2	Nonsynonymous:GCKR	C	T	0,021	0,002		-0,006	0,009	0,464
rs13024996	2	Intron:ARHGAP15	C	A	0,011	0,002		0,012	0,009	0,176
rs13032049	2	Intron:WDPCP	G	A	0,010	0,002		-0,012	0,009	0,185
rs13383034	2	Intron:LINC01833	T	C	0,015	0,002		-0,018	0,009	0,037
rs2178197	2	Intron:GPN1	A	G	0,009	0,002		0,009	0,009	0,306
rs56337305	2	Intergenic	T	C	0,010	0,002		0,003	0,008	0,716
rs72859280	2	Intergenic	T	G	0,023	0,004		-0,020	0,027	0,463
rs77165542	2	Intergenic	C	T	0,026	0,004		-0,019	0,032	0,565
rs828867	2	Utr3:TET3	A	G	0,009	0,002		0,000	0,008	0,986
rs13066454	3	Intergenic	C	T	0,009	0,002		0,003	0,008	0,712
rs13094887	3	Intergenic	A	T	0,010	0,002		0,021	0,009	0,028
rs2011092	3	Intron:ZBTB38	T	C	0,009	0,002		0,000	0,009	0,967
rs35894540	3	Intron:CADM2	T	C	0,013	0,002		0,013	0,008	0,124
rs60654199	3	Intron:RASA2	C	A	0,017	0,003		-0,018	0,014	0,190
rs62250685	3	Intron:CADM2	A	G	0,014	0,002		0,012	0,008	0,148
rs6787172	3	Intron:RSRC1	T	G	0,008	0,002		-0,009	0,008	0,262
rs9838144	3	Intron:CPNE4	G	C	0,010	0,002		-0,008	0,011	0,473

rs10004020	4	Intergenic	A	G	0,009	0,002		-0,010	0,009	0,279
rs10028756	4	Intergenic	G	A	0,019	0,002		-0,003	0,014	0,845
rs113909752	4	Intron:ADH1C	C	T	0,039	0,004		0,015	0,016	0,331
rs11940694	4	Intron:KLB	G	A	0,026	0,002		0,016	0,008	0,044
rs1229984	4	Nonsynonymous:ADH1B	C	T	0,151	0,004		0,043	0,017	0,014
rs12499107	4	Intergenic	G	A	0,013	0,002		0,014	0,011	0,216
rs12651313	4	Intergenic	C	G	0,009	0,002		0,010	0,008	0,230
rs13107325	4	Nonsynonymous:SLC39A8	C	T	0,028	0,003		0,001	0,019	0,980
rs17029090	4	Synonymous:C4orf17	A	G	0,049	0,005		-0,016	0,018	0,363
rs2165670	4	Intergenic	A	G	0,023	0,002		0,037	0,014	0,007
rs36052336	4	Intron:ADH1C	A	G	0,018	0,003		0,002	0,020	0,912
rs3748034	4	Nonsynonymous:HGFAC	G	T	0,012	0,002		-0,021	0,011	0,064
rs4501255	4	Intron:BEND4	G	C	0,011	0,002		-0,007	0,010	0,499
rs4690727	4	Intron:INPP4B	G	C	0,011	0,002		0,001	0,009	0,912
rs4699791	4	Intergenic	A	G	0,019	0,003		-0,001	0,013	0,926
rs79139602	4	Intron:C4orf17	T	A	0,060	0,005		-0,009	0,016	0,555
rs11739827	5	Intron:TENM2	G	T	0,008	0,002		0,005	0,009	0,564
rs12655091	5	Intergenic	G	A	0,008	0,002		-0,002	0,008	0,759
rs4916723	5	Intron:LINC00461	A	C	0,010	0,002		-0,008	0,008	0,306
rs55872084	5	Intron:SGCD	T	G	0,010	0,002		-0,005	0,010	0,630
rs10085696	7	Intron:AUTS2	A	G	0,011	0,002		-0,004	0,010	0,684
rs10236149	7	Intron:ARPC1B	A	G	0,014	0,002		0,004	0,011	0,698
rs35034355	7	Intron:ORC5	G	A	0,008	0,002		-0,011	0,008	0,192
rs6460047	7	Intergenic	C	T	0,012	0,002		-0,010	0,011	0,366
rs6951574	7	Intergenic	C	T	0,013	0,002		0,001	0,010	0,893
rs1217091	8	Intergenic	C	T	0,012	0,002		-0,013	0,011	0,243
rs13250583	8	Intergenic	C	T	0,010	0,002		-0,003	0,009	0,770
rs28601761	8	Intergenic	G	C	0,009	0,002		-0,005	0,008	0,519

rs10978550	9	Intergenic	T	C	0,012	0,002		0,015	0,010	0,130
rs55932213	9	Intergenic	G	A	0,010	0,002		0,015	0,009	0,101
rs17665139	10	Intergenic	C	T	0,012	0,002		0,003	0,012	0,822
rs7074871	10	Intergenic	G	A	0,009	0,002		0,006	0,009	0,479
rs10750025	11	Intergenic	T	C	0,010	0,002		0,010	0,009	0,257
rs11030084	11	Intron:BDNF-AS|LINC00678	C	T	0,011	0,002		0,012	0,010	0,222
rs12795042	11	Intron:LOC646522	A	C	0,008	0,002		-0,004	0,009	0,684
rs1713676	11	Intergenic	A	G	0,008	0,002		0,020	0,008	0,013
rs4938230	11	Intergenic	A	C	0,013	0,002		-0,016	0,011	0,148
rs56030824	11	Intron:SPI1	G	A	0,012	0,002		-0,010	0,008	0,217
rs682011	11	Intergenic	C	T	0,008	0,002		-0,002	0,008	0,829
rs7950166	11	Intron:TRIM66	C	T	0,010	0,002		0,000	0,008	0,998
rs10506274	12	Intron:ACSS3	G	T	0,009	0,002		0,001	0,008	0,867
rs10876188	12	Intron:SLC4A8	C	T	0,008	0,002		0,016	0,008	0,060
rs3809162	12	Intergenic	G	A	0,009	0,002		-0,022	0,008	0,007
rs4842786	12	Intergenic	G	A	0,009	0,002		-0,008	0,008	0,357
rs500321	13	Intergenic	A	T	0,010	0,002		0,006	0,010	0,565
rs1123285	14	Intron:OTX2	C	G	0,009	0,002		0,012	0,009	0,145
rs11625650	14	Intron:KIF26A	G	A	0,010	0,002		0,002	0,012	0,890
rs2180870	14	Intron:ARID4A	T	C	0,012	0,002		0,013	0,013	0,312
rs12907323	15	Intron:AGBL1	G	A	0,009	0,002		-0,002	0,008	0,829
rs2472297	15	Intergenic	T	C	0,011	0,002		0,000	0,011	0,982
rs1104608	16	Intergenic	G	C	0,011	0,002		-0,003	0,009	0,739
rs113443718	16	Intron:SEZ6L2	G	A	0,010	0,002		0,020	0,010	0,034
rs17177078	16	Intron:TNRC6A	C	T	0,022	0,003		0,013	0,019	0,475
rs2764771	16	Intergenic	A	G	0,010	0,002		0,004	0,008	0,667
rs378421	16	Intergenic	G	A	0,011	0,002		0,019	0,011	0,078
rs62044525	16	Intergenic	C	G	0,012	0,002		0,003	0,012	0,783

rs7185555	16	Intergenic	G	C	0,011	0,002		0,012	0,012	0,312
rs79616692	16	Intron:LINC01572	C	G	0,016	0,002		0,005	0,015	0,754
rs10438820	17	Intron:RPTOR	T	C	0,009	0,002		0,013	0,008	0,125
rs2854334	17	Intergenic	G	A	0,009	0,002		-0,001	0,008	0,899
rs3803800	17	Nonsynonymous:TNFSF12-TNFSF13|TNFSF13	G	A	0,011	0,002		-0,008	0,009	0,418
rs4548913	17	Intron:SRR	G	A	0,008	0,002		0,015	0,008	0,075
rs4092465	18	Intergenic	A	G	0,008	0,002		0,000	0,009	0,999
rs9950000	18	Intron:TCF4	C	T	0,009	0,002		0,014	0,008	0,077
rs281379	19	Intergenic	A	G	0,014	0,002		0,021	0,009	0,015
rs4815364	20	Intron:ACSS1	A	G	0,009	0,002		0,015	0,008	0,067
rs9607814	22	Intergenic	C	A	0,010	0,002		-0,003	0,011	0,792

*Data are from genome-wide association studies on alcohol consumption[1] and stroke.[4] †The betas for alcohol consumption are per SD increase of log-transformed drinks per week. The betas for stroke are the log odds ratios.

Table IV. Association between alcohol-related SNPs and intracerebral hemorrhage in the International Stroke Genetics Consortium*

					Association with alcohol				Association with intracerebral hemorrhage		
**SNP**	**Chr**	**Gene**	**EA**	**NEA**	**Beta†**	**SE**	**P**		**Beta†**	**SE**	**P**
rs10753661	1	Intergenic	G	A	0,009	0,002	3,76E-08		-0,068	0,055	0,218
rs12088813	1	Intron:PDE4B	A	C	0,009	0,002	1,58E-08		0,160	0,059	0,006
rs28680958	1	Intron:ZBTB37	G	A	0,011	0,002	5,13E-10		0,083	0,060	0,171
rs5024204	1	Intron:PTGER3	T	A	0,010	0,002	2,55E-09		0,004	0,057	0,939
rs58107686	1	Intron:PHC2	C	A	0,010	0,002	7,79E-10		0,049	0,054	0,365
rs705687	1	Intergenic	A	G	0,011	0,002	8,15E-10		-0,137	0,064	0,032
rs823114	1	Intergenic	A	G	0,009	0,002	2,31E-09		0,057	0,051	0,267
rs1260326	2	Nonsynonymous:GCKR	C	T	0,021	0,002	8,05E-45		0,004	0,052	0,946
rs13024996	2	Intron:ARHGAP15	C	A	0,011	0,002	5,72E-13		0,072	0,054	0,183
rs13032049	2	Intron:WDPCP	G	A	0,010	0,002	3,00E-10		0,050	0,056	0,378
rs17424787	2	Nonsynonymous:ACTR1B	A	G	0,017	0,003	2,53E-11		0,008	0,096	0,932
rs2178197	2	Intron:GPN1	A	G	0,009	0,002	2,45E-09		0,136	0,052	0,009
rs72859280	2	Intergenic	T	G	0,023	0,004	4,44E-09		-0,110	0,152	0,470
rs7594223	2	Intergenic	T	C	0,010	0,002	1,63E-10		0,016	0,053	0,755
rs13066454	3	Intergenic	C	T	0,009	0,002	4,13E-09		0,037	0,052	0,477
rs2011092	3	Intron:ZBTB38	T	C	0,009	0,002	7,35E-09		0,017	0,054	0,756
rs35012514	3	Intergenic	A	T	0,010	0,002	8,57E-11		-0,097	0,054	0,072
rs35894540	3	Intron:CADM2	T	C	0,013	0,002	1,58E-14		-0,066	0,053	0,208
rs60654199	3	Intron:RASA2	C	A	0,017	0,003	2,85E-08		-0,200	0,120	0,095
rs62250685	3	Intron:CADM2	A	G	0,014	0,002	1,05E-21		-0,070	0,053	0,187
rs6787172	3	Intron:RSRC1	T	G	0,008	0,002	4,27E-08		-0,005	0,052	0,925
rs9858200	3	Intron:CPNE4	G	A	0,010	0,002	2,65E-08		0,064	0,061	0,293
rs10004020	4	Intergenic	A	G	0,009	0,002	2,43E-08		0,020	0,056	0,728
rs10028756	4	Intergenic	G	A	0,019	0,002	1,16E-17		0,008	0,076	0,918
rs11097708	4	Intergenic	T	C	0,019	0,003	6,58E-14		-0,051	0,089	0,570
rs113909752	4	Intron:ADH1C	C	T	0,039	0,004	6,56E-27		0,192	0,104	0,064

rs11940694	4	Intron:KLB	G	A	0,026	0,002	3,03E-68		0,064	0,053	0,230
rs12499107	4	Intergenic	G	A	0,013	0,002	4,45E-09		0,001	0,079	0,994
rs12651313	4	Intergenic	C	G	0,009	0,002	3,79E-09		0,049	0,052	0,345
rs17029090	4	Synonymous:C4orf17	A	G	0,049	0,005	4,75E-21		0,351	0,193	0,068
rs2165670	4	Intergenic	A	G	0,023	0,002	1,67E-22		0,020	0,084	0,809
rs36052336	4	Intron:ADH1C	A	G	0,018	0,003	1,23E-09		0,141	0,113	0,212
rs4501255	4	Intron:BEND4	G	C	0,011	0,002	4,83E-10		-0,043	0,060	0,478
rs4690727	4	Intron:INPP4B	G	C	0,011	0,002	2,43E-11		-0,052	0,057	0,367
rs79139602	4	Intron:C4orf17	T	A	0,060	0,005	1,80E-32		-0,523	0,181	0,004
rs11739827	5	Intron:TENM2	G	T	0,008	0,002	1,18E-08		-0,008	0,052	0,881
rs12655091	5	Intergenic	G	A	0,008	0,002	1,25E-08		0,015	0,052	0,778
rs17422060	5	Intron:LINC00461	T	G	0,010	0,002	1,72E-11		-0,036	0,053	0,498
rs55872084	5	Intron:SGCD	T	G	0,010	0,002	6,32E-09		0,015	0,061	0,803
rs10085696	7	Intron:AUTS2	A	G	0,011	0,002	1,12E-09		0,071	0,067	0,286
rs10236149	7	Intron:ARPC1B	A	G	0,014	0,002	1,18E-09		-0,101	0,080	0,203
rs2533125	7	Intergenic	G	A	0,013	0,002	1,58E-19		0,022	0,052	0,677
rs35034355	7	Intron:ORC5	G	A	0,008	0,002	2,87E-08		0,061	0,051	0,233
rs6460047	7	Intergenic	C	T	0,012	0,002	9,69E-11		-0,012	0,063	0,852
rs1217091	8	Intergenic	C	T	0,012	0,002	7,05E-11		0,014	0,065	0,833
rs13250583	8	Intergenic	C	T	0,010	0,002	4.70E-08		-0,033	0,063	0,603
rs28601761	8	Intergenic	G	C	0,009	0,002	7,17E-10		-0,060	0,053	0,262
rs10978550	9	Intergenic	T	C	0,012	0,002	7,15E-11		-0,013	0,062	0,833
rs17665139	10	Intergenic	C	T	0,012	0,002	1,59E-08		0,106	0,072	0,143
rs7074871	10	Intergenic	G	A	0,009	0,002	1,86E-08		0,033	0,060	0,575
rs11030084	11	Intron:BDNF-AS|LINC00678	C	T	0,011	0,002	1,72E-08		-0,014	0,067	0,838
rs1713676	11	Intergenic	A	G	0,008	0,002	4,29E-08		-0,009	0,051	0,856
rs4309187	11	Intergenic	C	A	0,010	0,002	4,89E-11		0,043	0,056	0,437
rs4938230	11	Intergenic	A	C	0,013	0,002	1,48E-10		-0,090	0,068	0,188

rs530647	11	Intergenic	G	C	0,008	0,002	2,22E-08		0,107	0,052	0,039
rs56030824	11	Intron:SPI1	G	A	0,012	0,002	1,15E-13		-0,044	0,055	0,418
rs7950166	11	Intron:TRIM66	C	T	0,010	0,002	9,89E-11		0,061	0,054	0,253
rs10506274	12	Intron:ACSS3	G	T	0,009	0,002	5,78E-10		0,087	0,051	0,087
rs10876188	12	Intron:SLC4A8	C	T	0,008	0,002	4,84E-08		-0,030	0,051	0,554
rs3809162	12	Intergenic	G	A	0,009	0,002	1,19E-09		0,040	0,053	0,455
rs4842786	12	Intergenic	G	A	0,009	0,002	2,73E-09		-0,019	0,052	0,720
rs574593	13	Intergenic	T	C	0,010	0,002	4,92E-09		-0,151	0,058	0,009
rs2180870	14	Intron:ARID4A	T	C	0,012	0,002	1,12E-08		0,223	0,077	0,004
rs28929474	14	Nonsynonymous:SERPINA1	C	T	0,037	0,005	1,34E-11		-0,002	0,187	0,992
rs962961	14	Intron:OTX2	C	T	0,009	0,002	8,14E-09		0,033	0,055	0,552
rs12907323	15	Intron:AGBL1	G	A	0,009	0,002	9,93E-09		0,002	0,052	0,966
rs2472297	15	Intergenic	T	C	0,011	0,002	3,1E-10		0,071	0,063	0,259
rs11641912	16	Intergenic	A	G	0,011	0,002	4,24E-08		0,177	0,071	0,013
rs12930239	16	Intron:SEZ6L2	C	G	0,010	0,002	1,19E-10		-0,056	0,055	0,306
rs17177078	16	Intron:TNRC6A	C	T	0,022	0,003	1,27E-13		0,109	0,102	0,286
rs2764771	16	Intergenic	A	G	0,010	0,002	4,02E-10		0,024	0,056	0,663
rs35626515	16	Intergenic	C	A	0,011	0,002	4,83E-14		0,102	0,054	0,058
rs62044525	16	Intergenic	C	G	0,012	0,002	1,03E-10		-0,042	0,066	0,528
rs79616692	16	Intron:LINC01572	C	G	0,016	0,002	4,11E-12		0,012	0,083	0,884
rs10438820	17	Intron:RPTOR	T	C	0,009	0,002	1,76E-08		0,083	0,058	0,149
rs216197	17	Intron:SRR	A	G	0,008	0,002	3,11E-08		0,037	0,054	0,489
rs2854334	17	Intergenic	G	A	0,009	0,002	7,51E-10		0,006	0,053	0,908
rs9950000	18	Intron:TCF4	C	T	0,009	0,002	9,38E-10		0,023	0,052	0,663
rs281379	19	Intergenic	A	G	0,014	0,002	4,91E-21		-0,014	0,052	0,783
rs2387577	20	Intron:ACSS1	C	G	0,009	0,002	1,02E-08		0,042	0,055	0,445
rs9607814	22	Intergenic	C	A	0,010	0,002	4,31E-08		-0,031	0,065	0,631

*Data are from genome-wide association studies on alcohol consumption[1] and intracerebral hemorrhage.[5] †The betas for alcohol consumption are per SD increase of log transformed drinks per week. The betas for intracerebral hemorrhage are log odds ratios.

Table V. Association between alcohol-related SNPs and coronary artery disease in the CARDIoGRAMplusC4D consortium*

					Associations with alcohol				Associations with CAD		
**SNP**	**Chr**	**Gene**	**EA**	**NEA**	**Beta†**	**SE**	**P**		**Beta†**	**SE**	**P**
rs10753661	1	Intergenic	G	A	0,009	0,002	3,76E-08		0,015	0,011	0,174
rs12088813	1	Intron:PDE4B	A	C	0,009	0,002	1,58E-08		0,021	0,010	0,039
rs28680958	1	Intron:ZBTB37	G	A	0,011	0,002	5,13E-10		-0,004	0,011	0,744
rs5024204	1	Intron:PTGER3	T	A	0,010	0,002	2,55E-09		-0,011	0,010	0,267
rs58107686	1	Intron:PHC2	C	A	0,010	0,002	7,79E-10		-0,001	0,010	0,897
rs705687	1	Intergenic	A	G	0,011	0,002	8,15E-10		0,002	0,011	0,830
rs823114	1	Intergenic	A	G	0,009	0,002	2,31E-09		-0,010	0,009	0,262
rs11692435	2	Nonsynonymous:ACTR1B	A	G	0,017	0,003	2,53E-11		0,024	0,020	0,218
rs1260326	2	Nonsynonymous:GCKR	C	T	0,021	0,002	8,05E-45		0,003	0,010	0,735
rs13024996	2	Intron:ARHGAP15	C	A	0,011	0,002	5,72E-13		0,001	0,011	0,933
rs13032049	2	Intron:WDPCP	G	A	0,010	0,002	3,00E-10		-0,019	0,011	0,069
rs13383034	2	Intron:LINC01833	T	C	0,015	0,002	6,31E-22		-0,002	0,010	0,813
rs2178197	2	Intron:GPN1	A	G	0,009	0,002	2,45E-09		0,001	0,009	0,942
rs56337305	2	Intergenic	T	C	0,010	0,002	1,63E-10		0,013	0,010	0,206
rs72859280	2	Intergenic	T	G	0,023	0,004	4,44E-09		0,028	0,028	0,317
rs77165542	2	Intergenic	C	T	0,026	0,004	5,63E-11		0,060	0,041	0,146
rs828867	2	Utr3:TET3	A	G	0,009	0,002	2,15E-09		0,003	0,010	0,758
rs13066454	3	Intergenic	C	T	0,009	0,002	4,13E-09		-0,005	0,010	0,576
rs13094887	3	Intergenic	A	T	0,010	0,002	8,57E-11		0,021	0,011	0,057
rs2011092	3	Intron:ZBTB38	T	C	0,009	0,002	7,35E-09		0,019	0,011	0,078
rs35894540	3	Intron:CADM2	T	C	0,013	0,002	1,58E-14		-0,002	0,010	0,818
rs60654199	3	Intron:RASA2	C	A	0,017	0,003	2,85E-08		-0,027	0,018	0,134
rs62250685	3	Intron:CADM2	A	G	0,014	0,002	1,05E-21		-0,003	0,010	0,741
rs6787172	3	Intron:RSRC1	T	G	0,008	0,002	4,27E-08		-0,001	0,009	0,936
rs9838144	3	Intron:CPNE4	G	C	0,010	0,002	2,65E-08		-0,014	0,012	0,270

rs10004020	4	Intergenic	A	G	0,009	0,002	2,43E-08		0,000	0,010	0,971
rs10028756	4	Intergenic	G	A	0,019	0,002	1,16E-17		-0,016	0,014	0,251
rs113909752	4	Intron:ADH1C	C	T	0,039	0,004	6,56E-27		0,014	0,018	0,430
rs11940694	4	Intron:KLB	G	A	0,026	0,002	3,03E-68		0,012	0,009	0,204
rs1229984	4	Nonsynonymous:ADH1B	C	T	0,151	0,004	<2,2e-308		0,033	0,019	0,075
rs12499107	4	Intergenic	G	A	0,013	0,002	4,45E-09		-0,012	0,013	0,345
rs12651313	4	Intergenic	C	G	0,009	0,002	3,79E-09		0,005	0,010	0,637
rs13107325	4	Nonsynonymous:SLC39A8	C	T	0,028	0,003	1,53E-22		0,007	0,022	0,765
rs17029090	4	Synonymous:C4orf17	A	G	0,049	0,005	4,75E-21		-0,049	0,025	0,045
rs2165670	4	Intergenic	A	G	0,023	0,002	1,67E-22		0,016	0,015	0,293
rs36052336	4	Intron:ADH1C	A	G	0,018	0,003	1,23E-09		-0,005	0,022	0,823
rs3748034	4	Nonsynonymous:HGFAC	G	T	0,012	0,002	1,67E-08		-0,001	0,014	0,954
rs4501255	4	Intron:BEND4	G	C	0,011	0,002	4,83E-10		0,011	0,012	0,365
rs4690727	4	Intron:INPP4B	G	C	0,011	0,002	2,43E-11		-0,002	0,010	0,820
rs4699791	4	Intergenic	A	G	0,019	0,003	6,58E-14		0,013	0,015	0,377
rs79139602	4	Intron:C4orf17	T	A	0,060	0,005	1,80E-32		0,047	0,022	0,035
rs11739827	5	Intron:TENM2	G	T	0,008	0,002	1,18E-08		-0,010	0,009	0,295
rs12655091	5	Intergenic	G	A	0,008	0,002	1,25E-08		-0,016	0,009	0,083
rs4916723	5	Intron:LINC00461	A	C	0,010	0,002	1,72E-11		-0,018	0,010	0,068
rs55872084	5	Intron:SGCD	T	G	0,010	0,002	6,32E-09		-0,010	0,011	0,401
rs10085696	7	Intron:AUTS2	A	G	0,011	0,002	1,12E-09		-,0090	0,011	0,431
rs10236149	7	Intron:ARPC1B	A	G	0,014	0,002	1,18E-09		0,013	0,013	0,303
rs35034355	7	Intron:ORC5	G	A	0,008	0,002	2,87E-08		-0,001	0,009	0,895
rs6460047	7	Intergenic	C	T	0,012	0,002	9,69E-11		-0,002	0,012	0,867
rs6951574	7	Intergenic	C	T	0,013	0,002	1,58E-19		0,015	0,012	0,225
rs1217091	8	Intergenic	C	T	0,012	0,002	7,05E-11		0,008	0,013	0,515
rs13250583	8	Intergenic	C	T	0,010	0,002	4,70E-8		0,001	0,011	0,950
rs28601761	8	Intergenic	G	C	0,009	0,002	7,17E-10		-0,048	0,010	0,000

rs10978550	9	Intergenic	T	C	0,012	0,002	7,15E-11		0,013	0,012	0,277
rs55932213	9	Intergenic	G	A	0,010	0,002	9,55E-09		0,004	0,011	0,692
rs17665139	10	Intergenic	C	T	0,012	0,002	1,59E-08		-0,010	0,015	0,492
rs7074871	10	Intergenic	G	A	0,009	0,002	1,86E-08		-0,009	0,011	0,372
rs10750025	11	Intergenic	T	C	0,010	0,002	4,89E-11		0,003	0,011	0,791
rs11030084	11	Intron:BDNF-AS|LINC00678	C	T	0,011	0,002	1,72E-08		0,031	0,012	0,008
rs12795042	11	Intron:LOC646522	A	C	0,008	0,002	3,25E-08		0,006	0,010	0,556
rs1713676	11	Intergenic	A	G	0,008	0,002	4,29E-08		0,006	0,009	0,542
rs4938230	11	Intergenic	A	C	0,013	0,002	1,48E-10		0,009	0,012	0,442
rs56030824	11	Intron:SPI1	G	A	0,012	0,002	1,15E-13		0,008	0,010	0,416
rs682011	11	Intergenic	C	T	0,008	0,002	2,22E-08		-0,002	0,010	0,829
rs7950166	11	Intron:TRIM66	C	T	0,010	0,002	9,89E-11		-0,015	0,010	0,114
rs10506274	12	Intron:ACSS3	G	T	0,009	0,002	5,78E-10		-0,017	0,009	0,074
rs10876188	12	Intron:SLC4A8	C	T	0,008	0,002	4,84E-08		0,007	0,010	0,450
rs3809162	12	Intergenic	G	A	0,009	0,002	1,19E-09		-0,016	0,010	0,090
rs4842786	12	Intergenic	G	A	0,009	0,002	2,73E-09		-0,001	0,010	0,892
rs500321	13	Intergenic	A	T	0,010	0,002	4,92E-09		0,010	0,011	0,385
rs1123285	14	Intron:OTX2	C	G	0,009	0,002	8,14E-09		0,006	0,010	0,585
rs11625650	14	Intron:KIF26A	G	A	0,010	0,002	2,89E-08		0,016	0,014	0,259
rs2180870	14	Intron:ARID4A	T	C	0,012	0,002	1,12E-08		0,007	0,014	0,631
rs28929474	14	Nonsynonymous:SERPINA1	C	T	0,037	0,005	1,34E-11		0,147	0,045	0,001
rs12907323	15	Intron:AGBL1	G	A	0,009	0,002	9,93E-09		0,005	0,009	0,632
rs2472297	15	Intergenic	T	C	0,011	0,002	3,1E-10		-0,003	0,013	0,818
rs1104608	16	Intergenic	G	C	0,011	0,002	1,05E-13		-0,002	0,010	0,816
rs113443718	16	Intron:SEZ6L2	G	A	0,010	0,002	1,19E-10		0,003	0,012	0,793
rs17177078	16	Intron:TNRC6A	C	T	0,022	0,003	1,27E-13		0,016	0,021	0,453
rs2764771	16	Intergenic	A	G	0,010	0,002	4,02E-10		-0,011	0,010	0,248
rs378421	16	Intergenic	G	A	0,011	0,002	4,83E-14		-0,012	0,013	0,347

rs62044525	16	Intergenic	C	G	0,012	0,002	1,03E-10		-0,011	0,013	0,386
rs7185555	16	Intergenic	G	C	0,011	0,002	4,24E-08		0,002	0,014	0,891
rs79616692	16	Intron:LINC01572	C	G	0,016	0,002	4,11E-12		-0,031	0,016	0,055
rs10438820	17	Intron:RPTOR	T	C	0,009	0,002	1,76E-08		-0,016	0,010	0,108
rs2854334	17	Intergenic	G	A	0,009	0,002	7,51E-10		-0,010	0,010	0,298
rs3803800	17	Nonsynonymous:TNFSF12-13	G	A	0,011	0,002	1,5E-10		-0,013	0,011	0,240
rs4548913	17	Intron:SRR	G	A	0,008	0,002	3,11E-08		0,037	0,010	0,000
rs4092465	18	Intergenic	A	G	0,008	0,002	4,39E-08		0,005	0,010	0,648
rs9950000	18	Intron:TCF4	C	T	0,009	0,002	9,38E-10		-0,005	0,009	0,632
rs281379	19	Intergenic	A	G	0,014	0,002	4,91E-21		0,011	0,010	0,298
rs4815364	20	Intron:ACSS1	A	G	0,009	0,002	1,02E-08		0,012	0,010	0,233
rs9607814	22	Intergenic	C	A	0,010	0,002	4,31E-08		0,006	0,011	0,608

*Data are from genome-wide association studies on alcohol consumption[1] and coronary artery disease.[16] † The betas for alcohol consumption are per SD increase of log-transformed drinks per week. The betas for coronary artery disease are log odds ratios.

Table VI. Association between alcohol-related SNPs and atrial fibrillation in the Atrial Fibrillation Consortium*

					Associations with alcohol				Association with atrial fibrillation		
SNP	Chr	Gene	EA	NEA	Beta†	SE	P		Beta†	SE	P
rs10753661	1	Intergenic	G	A	0,009	0,002	3,76E-08		0,007	0,008	0,368
rs12088813	1	Intron:PDE4B	A	C	0,009	0,002	1,58E-08		0,007	0,008	0,401
rs28680958	1	Intron:ZBTB37	G	A	0,011	0,002	5,13E-10		0,006	0,009	0,516
rs5024204	1	Intron:PTGER3	T	A	0,010	0,002	2,55E-09		0,003	0,008	0,753
rs58107686	1	Intron:PHC2	C	A	0,010	0,002	7,79E-10		0,012	0,008	0,123
rs705687	1	Intergenic	A	G	0,011	0,002	8,15E-10		0,029	0,009	0,001
rs823114	1	Intergenic	A	G	0,009	0,002	2,31E-09		-0,037	0,007	0,000
rs11692435	2	Nonsynonymous:ACTR1B	A	G	0,017	0,003	2,53E-11		0,011	0,014	0,401
rs1260326	2	Nonsynonymous:GCKR	C	T	0,021	0,002	8,05E-45		0,007	0,007	0,340
rs13024996	2	Intron:ARHGAP15	C	A	0,011	0,002	5,72E-13		-0,002	0,008	0,828
rs13032049	2	Intron:WDPCP	G	A	0,010	0,002	3E-10		0,008	0,008	0,315
rs13383034	2	Intron:LINC01833	T	C	0,015	0,002	6,31E-22		0,002	0,008	0,772
rs2178197	2	Intron:GPN1	A	G	0,009	0,002	2,45E-09		-0,003	0,007	0,705
rs56337305	2	Intergenic	T	C	0,010	0,002	1,63E-10		0,003	0,007	0,691
rs72859280	2	Intergenic	T	G	0,023	0,004	4,44E-09		0,039	0,021	0,067
rs77165542	2	Intergenic	C	T	0,026	0,004	5,63E-11		0,050	0,025	0,043
rs828867	2	Utr3:TET3	A	G	0,009	0,002	2,15E-09		0,015	0,007	0,035
rs13066454	3	Intergenic	C	T	0,009	0,002	4,13E-09		-0,007	0,007	0,314
rs13094887	3	Intergenic	A	T	0,010	0,002	8,57E-11		-0,008	0,008	0,308
rs2011092	3	Intron:ZBTB38	T	C	0,009	0,002	7,35E-09		-0,022	0,008	0,006
rs35894540	3	Intron:CADM2	T	C	0,013	0,002	1,58E-14		0,013	0,007	0,087
rs60654199	3	Intron:RASA2	C	A	0,017	0,003	2,85E-08		-0,025	0,013	0,063
rs62250685	3	Intron:CADM2	A	G	0,014	0,002	1,05E-21		0,012	0,007	0,096
rs6787172	3	Intron:RSRC1	T	G	0,008	0,002	4,27E-08		-0,006	0,007	0,406
rs9838144	3	Intron:CPNE4	G	C	0,010	0,002	2,65E-08		-0,017	0,009	0,068

rs10004020	4	Intergenic	A	G	0,009	0,002	2,43E-08		-0,004	0,008	0,582
rs10028756	4	Intergenic	G	A	0,019	0,002	1,16E-17		-0,008	0,011	0,486
rs113909752	4	Intron:ADH1C	C	T	0,039	0,004	6,56E-27		0,030	0,014	0,035
rs11940694	4	Intron:KLB	G	A	0,026	0,002	3,03E-68		0,014	0,007	0,049
rs1229984	4	Nonsynonymous:ADH1B	C	T	0,151	0,004	<2,2e-308		0,023	0,019	0,216
rs12499107	4	Intergenic	G	A	0,013	0,002	4,45E-09		0,001	0,010	0,902
rs12651313	4	Intergenic	C	G	0,009	0,002	3,79E-09		0,012	0,007	0,101
rs13107325	4	Nonsynonymous:SLC39A8	C	T	0,028	0,003	1,53E-22		0,000	0,015	0,986
rs17029090	4	Synonymous:C4orf17	A	G	0,049	0,005	4,75E-21		-0,005	0,020	0,806
rs2165670	4	Intergenic	A	G	0,023	0,002	1,67E-22		0,024	0,012	0,044
rs36052336	4	Intron:ADH1C	A	G	0,018	0,003	1,23E-09		-0,002	0,016	0,905
rs3748034	4	Nonsynonymous:HGFAC	G	T	0,012	0,002	1,67E-08		0,013	0,010	0,210
rs4501255	4	Intron:BEND4	G	C	0,011	0,002	4,83E-10		0,000	0,009	0,962
rs4690727	4	Intron:INPP4B	G	C	0,011	0,002	2,43E-11		0,001	0,008	0,942
rs4699791	4	Intergenic	A	G	0,019	0,003	6,58E-14		0,009	0,012	0,440
rs7682824	4	Intergenic	T	C	0,008	0,002	2,77E-08		0,004	0,013	0,740
rs79139602	4	Intron:C4orf17	T	A	0,060	0,005	1,80E-32		-0,019	0,018	0,272
rs11739827	5	Intron:TENM2	G	T	0,008	0,002	1,18E-08		0,000	0,007	0,969
rs12655091	5	Intergenic	G	A	0,008	0,002	1,25E-08		-0,005	0,007	0,488
rs4916723	5	Intron:LINC00461	A	C	0,010	0,002	1,72E-11		-0,003	0,007	0,663
rs55872084	5	Intron:SGCD	T	G	0,010	0,002	6,32E-09		-0,008	0,009	0,369
rs10085696	7	Intron:AUTS2	A	G	0,011	0,002	1,12E-09		0,011	0,009	0,228
rs10236149	7	Intron:ARPC1B	A	G	0,014	0,002	1,18E-09		-0,009	0,010	0,399
rs35034355	7	Intron:ORC5	G	A	0,008	0,002	2,87E-08		-0,006	0,007	0,380
rs6460047	7	Intergenic	C	T	0,012	0,002	9,69E-11		0,015	0,009	0,098
rs6951574	7	Intergenic	C	T	0,013	0,002	1,58E-19		-0,005	0,008	0,538
rs1217091	8	Intergenic	C	T	0,012	0,002	7,05E-11		-0,011	0,009	0,251
rs13250583	8	Intergenic	C	T	0,010	0,002	4,70E-8		0,017	0,009	0,055

rs28601761	8	Intergenic	G	C	0,009	0,002	7,17E-10		-0,001	0,007	0,879
rs10978550	9	Intergenic	T	C	0,012	0,002	7,15E-11		-0,001	0,009	0,887
rs55932213	9	Intergenic	G	A	0,010	0,002	9,55E-09		0,017	0,008	0,036
rs17665139	10	Intergenic	C	T	0,012	0,002	1,59E-08		0,003	0,010	0,786
rs7074871	10	Intergenic	G	A	0,009	0,002	1,86E-08		0,008	0,008	0,304
rs10750025	11	Intergenic	T	C	0,010	0,002	4,89E-11		0,000	0,008	0,955
rs11030084	11	Intron:BDNF-AS|LINC00678	C	T	0,011	0,002	1,72E-08		-0,001	0,009	0,909
rs12795042	11	Intron:LOC646522	A	C	0,008	0,002	3,25E-08		-0,008	0,008	0,270
rs1713676	11	Intergenic	A	G	0,008	0,002	4,29E-08		0,013	0,007	0,065
rs4938230	11	Intergenic	A	C	0,013	0,002	1,48E-10		0,010	0,010	0,297
rs56030824	11	Intron:SPI1	G	A	0,012	0,002	1,15E-13		-0,007	0,008	0,347
rs682011	11	Intergenic	C	T	0,008	0,002	2,22E-08		0,005	0,007	0,527
rs7950166	11	Intron:TRIM66	C	T	0,010	0,002	9,89E-11		0,001	0,007	0,934
rs10506274	12	Intron:ACSS3	G	T	0,009	0,002	5,78E-10		0,000	0,007	0,988
rs10876188	12	Intron:SLC4A8	C	T	0,008	0,002	4,84E-08		0,000	0,007	0,993
rs3809162	12	Intergenic	G	A	0,009	0,002	1,19E-09		-0,014	0,007	0,051
rs4842786	12	Intergenic	G	A	0,009	0,002	2,73E-09		0,008	0,007	0,254
rs500321	13	Intergenic	A	T	0,010	0,002	4,92E-09		-0,010	0,008	0,249
rs1123285	14	Intron:OTX2	C	G	0,009	0,002	8,14E-09		-0,004	0,008	0,563
rs11625650	14	Intron:KIF26A	G	A	0,010	0,002	2,89E-08		-0,005	0,009	0,584
rs2180870	14	Intron:ARID4A	T	C	0,012	0,002	1,12E-08		0,015	0,011	0,181
rs28929474	14	Nonsynonymous:SERPINA1	C	T	0,037	0,005	1,34E-11		-0,001	0,031	0,973
rs12907323	15	Intron:AGBL1	G	A	0,009	0,002	9,93E-09		0,004	0,007	0,595
rs2472297	15	Intergenic	T	C	0,011	0,002	3,1E-10		-0,002	0,009	0,865
rs1104608	16	Intergenic	G	C	0,011	0,002	1,05E-13		-0,002	0,008	0,813
rs113443718	16	Intron:SEZ6L2	G	A	0,010	0,002	1,19E-10		0,005	0,008	0,502
rs17177078	16	Intron:TNRC6A	C	T	0,022	0,003	1,27E-13		-0,010	0,016	0,548
rs2764771	16	Intergenic	A	G	0,010	0,002	4,02E-10		-0,003	0,008	0,718

rs378421	16	Intergenic	G	A	0,011	0,002	4,83E-14		0,000	0,008	0,991
rs62044525	16	Intergenic	C	G	0,012	0,002	1,03E-10		-0,004	0,010	0,717
rs7185555	16	Intergenic	G	C	0,011	0,002	4,24E-08		0,020	0,010	0,052
rs79616692	16	Intron:LINC01572	C	G	0,016	0,002	4,11E-12		0,006	0,012	0,641
rs10438820	17	Intron:RPTOR	T	C	0,009	0,002	1,76E-08		-0,001	0,008	0,858
rs2854334	17	Intergenic	G	A	0,009	0,002	7,51E-10		0,012	0,007	0,116
rs3803800	17	Nonsynonymous:TNFSF12-13	G	A	0,011	0,002	1,5E-10		-0,017	0,008	0,043
rs4548913	17	Intron:SRR	G	A	0,008	0,002	3,11E-08		0,017	0,008	0,024
rs4092465	18	Intergenic	A	G	0,008	0,002	4,39E-08		0,007	0,008	0,335
rs9950000	18	Intron:TCF4	C	T	0,009	0,002	9,38E-10		-0,005	0,007	0,473
rs281379	19	Intergenic	A	G	0,014	0,002	4,91E-21		-0,002	0,008	0,766
rs4815364	20	Intron:ACSS1	A	G	0,009	0,002	1,02E-08		0,010	0,008	0,183
rs9607814	22	Intergenic	C	A	0,010	0,002	4,31E-08		-0,013	0,009	0,180

*Data are from genome-wide association studies on alcohol consumption[1] and atrial fibrillation.[7] †The betas for alcohol consumption are per SD increase of log-transformed drinks per week. The betas for atrial fibrillation are log odds ratios.

Table VII. Associations between alcohol-related SNPs and cardiovascular diseases in UK Biobank*

					Alcohol consumption			Stroke			Coronary artery disease			Heart failure		
**SNP**	**Chr**	**Gene**	**EA**	**NEA**	**Beta†**	**SE**	**P**	**Beta†**	**SE**	**P**	**Beta†**	**SE**	**P**	**Beta†**	**SE**	**P**
rs10753661	1	Intergenic	G	A	0,009	0,002	3,76E-08	-0,007	0,016	0,672	-0,004	0,010	0,709	0,017	0,019	0,360
rs12088813	1	Intron:PDE4B	A	C	0,009	0,002	1,58E-08	0,024	0,017	0,147	0,014	0,010	0,156	0,020	0,020	0,317
rs28680958	1	Intron:ZBTB37	G	A	0,011	0,002	5,13E-10	-0,005	0,018	0,786	0,006	0,011	0,612	0,030	0,022	0,167
rs5024204	1	Intron:PTGER3	T	A	0,010	0,002	2,55E-09	-0,006	0,017	0,708	0,008	0,010	0,404	0,021	0,020	0,298
rs58107686	1	Intron:PHC2	C	A	0,010	0,002	7,79E-10	0,004	0,016	0,793	0,006	0,010	0,532	-0,006	0,019	0,738
rs705687	1	Intergenic	A	G	0,011	0,002	8,15E-10	0,006	0,018	0,724	-0,002	0,011	0,889	-0,020	0,021	0,357
rs823114	1	Intergenic	A	G	0,009	0,002	2,31E-09	0,006	0,015	0,686	-0,002	0,009	0,822	0,025	0,018	0,157
rs11692435	2	Nonsynonymous:ACTR1B	A	G	0,017	0,003	2,53E-11	-0,016	0,028	0,567	0,039	0,017	0,019	0,015	0,033	0,657
rs1260326	2	Nonsynonymous:GCKR	C	T	0,021	0,002	8,05E-45	0,000	0,015	0,985	-0,028	0,009	0,002	-0,016	0,018	0,370
rs13024996	2	Intron:ARHGAP15	C	A	0,011	0,002	5,72E-13	0,027	0,015	0,076	-0,007	0,009	0,433	0,035	0,018	0,057
rs13032049	2	Intron:WDPCP	G	A	0,010	0,002	3E-10	-0,005	0,016	0,767	-0,031	0,010	0,002	-0,019	0,020	0,330
rs13383034	2	Intron:LINC01833	T	C	0,015	0,002	6,31E-22	0,019	0,016	0,230	0,007	0,010	0,483	0,029	0,019	0,127
rs2178197	2	Intron:GPN1	A	G	0,009	0,002	2,45E-09	-0,015	0,015	0,319	0,002	0,009	0,806	0,003	0,018	0,883
rs56337305	2	Intergenic	T	C	0,010	0,002	1,63E-10	0,003	0,015	0,856	0,004	0,009	0,634	-0,038	0,018	0,038
rs72859280	2	Intergenic	T	G	0,023	0,004	4,44E-09	0,010	0,039	0,809	0,053	0,023	0,025	0,045	0,046	0,335
rs77165542	2	Intergenic	C	T	0,026	0,004	5,63E-11	-0,005	0,040	0,900	0,066	0,025	0,008	0,129	0,051	0,010
rs828867	2	Utr3:TET3	A	G	0,009	0,002	2,15E-09	-0,003	0,015	0,849	0,019	0,009	0,040	0,012	0,018	0,504
rs13066454	3	Intergenic	C	T	0,009	0,002	4,13E-09	-0,008	0,015	0,613	0,016	0,009	0,082	0,033	0,018	0,071
rs13094887	3	Intergenic	A	T	0,010	0,002	8,57E-11	-0,013	0,016	0,411	-0,012	0,010	0,214	-0,019	0,019	0,318
rs2011092	3	Intron:ZBTB38	T	C	0,009	0,002	7,35E-09	-0,020	0,015	0,206	0,010	0,009	0,291	-0,043	0,018	0,020
rs60654199	3	Intron:RASA2	C	A	0,017	0,003	2,85E-08	0,022	0,030	0,455	-0,012	0,018	0,484	0,005	0,035	0,890
rs62250685	3	Intron:CADM2	A	G	0,014	0,002	1,05E-21	0,019	0,015	0,201	0,010	0,009	0,286	0,004	0,018	0,809
rs6787172	3	Intron:RSRC1	T	G	0,008	0,002	4,27E-08	0,002	0,015	0,899	-0,012	0,009	0,179	-0,021	0,018	0,230

rs74664784	3	Intron:CADM2	T	C	0,013	0,002	1,58E-14		-0,203	0,360	0,585		0,206	0,257	0,410		-0,125	0,444	0,783
rs9838144	3	Intron:CPNE4	G	C	0,010	0,002	2,65E-08		0,005	0,018	0,765		0,011	0,011	0,332		0,002	0,022	0,937
rs10004020	4	Intergenic	A	G	0,009	0,002	2,43E-08		-0,029	0,016	0,072		0,002	0,010	0,845		-0,002	0,020	0,927
rs10028756	4	Intergenic	G	A	0,019	0,002	1,16E-17		-0,012	0,022	0,587		-0,011	0,013	0,407		-0,063	0,025	0,014
rs11940694	4	Intron:KLB	G	A	0,026	0,002	3,03E-68		0,001	0,015	0,951		0,006	0,009	0,496		0,018	0,018	0,321
rs1229984	4	Nonsynonymous:ADH1B	C	T	0,151	0,004	<2,2e-308		0,020	0,048	0,684		0,124	0,030	0,000		0,081	0,059	0,163
rs12499107	4	Intergenic	G	A	0,013	0,002	4,45E-09		0,015	0,022	0,484		-0,011	0,013	0,417		-0,002	0,026	0,947
rs12651313	4	Intergenic	C	G	0,009	0,002	3,79E-09		0,011	0,015	0,461		0,006	0,009	0,486		-0,011	0,018	0,532
rs13107325	4	Nonsynonymous:SLC39A8	C	T	0,028	0,003	1,53E-22		0,021	0,028	0,455		-0,005	0,017	0,788		-0,078	0,032	0,017
rs17029090	4	Synonymous:C4orf17	A	G	0,049	0,005	4,75E-21		-0,051	0,052	0,332		0,014	0,032	0,666		0,074	0,066	0,257
rs2165670	4	Intergenic	A	G	0,023	0,002	1,67E-22		0,034	0,024	0,161		0,037	0,015	0,012		0,020	0,029	0,491
rs36052336	4	Intron:ADH1C	A	G	0,018	0,003	1,23E-09		0,012	0,030	0,681		-0,008	0,018	0,673		-0,033	0,036	0,363
rs3748034	4	Nonsynonymous:HGFAC	G	T	0,012	0,002	1,67E-08		0,009	0,021	0,683		-0,036	0,013	0,004		-0,009	0,025	0,737
rs4501255	4	Intron:BEND4	G	C	0,011	0,002	4,83E-10		0,016	0,017	0,339		0,008	0,010	0,419		0,001	0,021	0,948
rs4690727	4	Intron:INPP4B	G	C	0,011	0,002	2,43E-11		0,003	0,017	0,881		-0,010	0,010	0,312		-0,023	0,020	0,248
rs4699791	4	Intergenic	A	G	0,019	0,003	6,58E-14		-0,026	0,025	0,301		0,003	0,015	0,832		-0,041	0,030	0,169
rs561222871	4	Intron:ADH1C	C	T	0,039	0,004	6,56E-27		0,387	0,604	0,503		0,301	0,348	0,374		0,384	0,727	0,581
rs7682824	4	Intergenic	T	C	0,008	0,002	2,77E-08		-,0300	0,021	0,152		0,008	0,013	0,514		-0,016	0,025	0,514
rs79139602	4	Intron:C4orf17	T	A	0,060	0,005	1,80E-32		0,031	0,051	0,553		-0,019	0,032	0,545		-0,086	0,065	0,180
rs11739827	5	Intron:TENM2	G	T	0,008	0,002	1,18E-08		-0,013	0,015	0,386		-0,001	0,009	0,940		0,004	0,018	0,845
rs12655091	5	Intergenic	G	A	0,008	0,002	1,25E-08		-0,011	0,015	0,458		-0,019	0,009	0,031		-0,009	0,018	0,621
rs4916723	5	Intron:LINC00461	A	C	0,010	0,002	1,72E-11		-0,012	0,015	0,443		0,006	0,009	0,529		0,012	0,018	0,506
rs55872084	5	Intron:SGCD	T	G	0,010	0,002	6,32E-09		-0,021	0,017	0,230		-0,004	0,010	0,739		-0,025	0,021	0,217
rs10085696	7	Intron:AUTS2	A	G	0,011	0,002	1,12E-09		0,003	0,019	0,889		0,013	0,012	0,268		0,049	0,023	0,033
rs10236149	7	Intron:ARPC1B	A	G	0,014	0,002	1,18E-09		0,009	0,022	0,703		0,025	0,014	0,070		0,039	0,027	0,146
rs35034355	7	Intron:ORC5	G	A	0,008	0,002	2,87E-08		0,004	0,015	0,802		-0,017	0,009	0,052		-0,020	0,018	0,256
rs6460047	7	Intergenic	C	T	0,012	0,002	9,69E-11		0,031	0,018	0,083		-0,002	0,011	0,830		-0,007	0,022	0,756

rs6951574	7	Intergenic	C	T	0,013	0,002	1,58E-19		0,014	0,015	0,350		0,014	0,009	0,118		-0,002	0,018	0,934
rs1217091	8	Intergenic	C	T	0,012	0,002	7,05E-11		0,039	0,019	0,040		0,001	0,011	0,935		-0,022	0,022	0,317
rs13250583	8	Intergenic	C	T	0,010	0,002	4,70E-08		-0,001	0,018	0,960		0,009	0,011	0,400		-0,007	0,022	0,739
rs28601761	8	Intergenic	G	C	0,009	0,002	7,17E-10		-0,015	0,015	0,334		-0,058	0,009	0,000		-0,058	0,018	0,001
rs10978550	9	Intergenic	T	C	0,012	0,002	7,15E-11		0,001	0,018	0,953		-0,011	0,011	0,313		0,046	0,022	0,036
rs55932213	9	Intergenic	G	A	0,010	0,002	9,55E-09		0,004	0,017	0,828		-0,004	0,010	0,708		-0,030	0,021	0,150
rs17665139	10	Intergenic	C	T	0,012	0,002	1,59E-08		-0,027	0,020	0,183		-0,010	0,012	0,404		-0,027	0,024	0,270
rs7074871	10	Intergenic	G	A	0,009	0,002	1,86E-08		0,004	0,017	0,794		0,000	0,010	0,990		-0,002	0,020	0,908
rs10750025	11	Intergenic	T	C	0,010	0,002	4,89E-11		0,026	0,016	0,108		0,011	0,010	0,244		0,015	0,019	0,429
rs11030084	11	Intron:BDNF	C	T	0,011	0,002	1,72E-08		0,026	0,019	0,169		0,007	0,012	0,532		-0,001	0,023	0,965
rs12795042	11	Intron:LOC646522	A	C	0,008	0,002	3,25E-08		-0,026	0,015	0,091		-0,015	0,009	0,103		-0,014	0,018	0,455
rs1713676	11	Intergenic	A	G	0,008	0,002	4,29E-08		0,011	0,015	0,476		0,026	0,009	0,003		0,018	0,018	0,312
rs4938230	11	Intergenic	A	C	0,013	0,002	1,48E-10		0,021	0,021	0,304		-0,015	0,012	0,216		0,024	0,025	0,335
rs56030824	11	Intron:SPI1	G	A	0,012	0,002	1,15E-13		0,008	0,016	0,617		0,007	0,010	0,436		-0,002	0,019	0,936
rs682011	11	Intergenic	C	T	0,008	0,002	2,22E-08		0,019	0,015	0,189		-0,001	0,009	0,878		-0,008	0,018	0,653
rs7950166	11	Intron:TRIM66	C	T	0,010	0,002	9,89E-11		0,019	0,015	0,213		-0,016	0,009	0,089		-0,011	0,018	0,566
rs10506274	12	Intron:ACSS3	G	T	0,009	0,002	5,78E-10		0,003	0,015	0,838		0,025	0,009	0,005		0,023	0,018	0,199
rs10876188	12	Intron:SLC4A8	C	T	0,008	0,002	4,84E-08		0,009	0,015	0,532		0,014	0,009	0,110		0,001	0,018	0,968
rs3809162	12	Intergenic	G	A	0,009	0,002	1,19E-09		-0,004	0,015	0,804		-0,017	0,009	0,065		0,011	0,018	0,524
rs4842786	12	Intergenic	G	A	0,009	0,002	2,73E-09		0,013	0,015	0,399		-0,010	0,009	0,275		0,003	0,018	0,878
rs500321	13	Intergenic	A	T	0,010	0,002	4,92E-09		0,030	0,016	0,066		0,001	0,010	0,917		-0,011	0,020	0,576
rs1123285	14	Intron:OTX2	C	G	0,009	0,002	8,14E-09		-0,017	0,016	0,286		0,004	0,010	0,715		0,007	0,019	0,705
rs11625650	14	Intron:KIF26A	G	A	0,010	0,002	2,89E-08		-0,012	0,017	0,493		0,026	0,011	0,012		0,009	0,021	0,679
rs2180870	14	Intron:ARID4A	T	C	0,012	0,002	1,12E-08		0,033	0,022	0,129		-0,026	0,013	0,048		-0,040	0,026	0,120
rs28929474	14	Nonsynonymous:SERPINA1	C	T	0,037	0,005	1,34E-11		-0,004	0,052	0,942		0,098	0,033	0,003		0,017	0,063	0,783
rs12907323	15	Intron:AGBL1	G	A	0,009	0,002	9,93E-09		0,003	0,015	0,823		0,003	0,009	0,758		0,013	0,018	0,483
rs2472297	15	Intergenic	T	C	0,011	0,002	3,1E-10		0,001	0,017	0,942		-0,018	0,010	0,083		-0,004	0,020	0,840

rs1104608	16	Intergenic	G	C	0,011	0,002	1,05E-13		0,007	0,015	0,632		0,009	0,009	0,309		-0,010	0,018	0,564
rs113443718	16	Intron:SEZ6L2	G	A	0,010	0,002	1,19E-10		-0,015	0,016	0,361		-0,018	0,010	0,068		-0,013	0,019	0,483
rs17177078	16	Intron:TNRC6A	C	T	0,022	0,003	1,27E-13		-0,038	0,032	0,236		-0,027	0,019	0,162		-0,034	0,038	0,367
rs2764771	16	Intergenic	A	G	0,010	0,002	4,02E-10		-0,008	0,016	0,636		-0,032	0,010	0,001		-0,048	0,019	0,013
rs378421	16	Intergenic	G	A	0,011	0,002	4,83E-14		0,001	0,015	0,924		0,005	0,009	0,564		-0,024	0,018	0,174
rs62044525	16	Intergenic	C	G	0,012	0,002	1,03E-10		-0,012	0,019	0,530		-0,008	0,011	0,509		0,024	0,023	0,283
rs7185555	16	Intergenic	G	C	0,011	0,002	4,24E-08		-0,001	0,020	0,970		0,033	0,012	0,007		0,027	0,024	0,273
rs79616692	16	Intron:LINC01572	C	G	0,016	0,002	4,11E-12		0,017	0,024	0,465		-0,007	0,015	0,616		-0,034	0,029	0,239
rs10438820	17	Intron:RPTOR	T	C	0,009	0,002	1,76E-08		-0,008	0,016	0,607		-0,009	0,010	0,381		0,036	0,019	0,064
rs2854334	17	Intergenic	G	A	0,009	0,002	7,51E-10		0,004	0,015	0,798		-0,002	0,009	0,871		-0,023	0,018	0,212
rs3803800	17	Nonsynonymous:TNFSF12	G	A	0,011	0,002	1,5E-10		0,032	0,018	0,081		0,003	0,011	0,815		0,000	0,022	0,991
rs4548913	17	Intron:SRR	G	A	0,008	0,002	3,11E-08		0,046	0,015	0,003		0,041	0,009	0,000		0,030	0,018	0,100
rs4092465	18	Intergenic	A	G	0,008	0,002	4,39E-08		0,001	0,016	0,970		-0,010	0,009	0,270		-0,019	0,019	0,295
rs9950000	18	Intron:TCF4	C	T	0,009	0,002	9,38E-10		-0,005	0,015	0,739		-0,009	0,009	0,352		-0,002	0,018	0,914
rs281379	19	Intergenic	A	G	0,014	0,002	4,91E-21		0,037	0,015	0,011		0,022	0,009	0,016		0,018	0,018	0,316
rs4815364	20	Intron:ACSS1	A	G	0,009	0,002	1,02E-08		-0,006	0,015	0,691		0,005	0,009	0,568		0,032	0,018	0,079
rs9607814	22	Intergenic	C	A	0,010	0,002	4,31E-08		-0,019	0,019	0,309		0,001	0,011	0,932		-0,016	0,022	0,471

*Data are from genome-wide association studies on alcohol consumption[1] and from UK Biobank. †The betas for alcohol consumption are per SD increase of log-transformed drinks per week and the betas for the cardiovascular diseases are log odds ratios.

Table VII continued. Association between alcohol-related SNPs and cardiovascular diseases in UK Biobank*

					Alcohol consumption			Venous thromboembolism			Peripheral artery disease			Aortic valve stenosis			Abdominal aortic aneurysm		
SNP	Chr	Gene	EA	NEA	Beta†	SE	P	Beta†	SE	P	Beta†	SE	P	Beta†	SE	P	Beta†	SE	P
rs10753661	1	Intergenic	G	A	0,009	0,002	3,76E-08	0,005	0,013	0,705	0,024	0,026	0,359	-0,014	0,033	0,669	-0,020	0,047	0,669
rs12088813	1	Intron:PDE4B	A	C	0,009	0,002	1,58E-08	0,029	0,014	0,034	0,005	0,027	0,844	-0,007	0,034	0,847	0,039	0,049	0,419
rs28680958	1	Intron:ZBTB37	G	A	0,011	0,002	5,13E-10	-0,025	0,015	0,096	0,023	0,030	0,432	-0,046	0,036	0,199	0,047	0,053	0,369
rs5024204	1	Intron:PTGER3	T	A	0,010	0,002	2,55E-09	0,002	0,014	0,894	0,006	0,028	0,824	0,052	0,034	0,126	-0,048	0,049	0,332
rs58107686	1	Intron:PHC2	C	A	0,010	0,002	7,79E-10	0,006	0,013	0,623	0,018	0,026	0,484	-0,038	0,032	0,232	0,032	0,046	0,490
rs705687	1	Intergenic	A	G	0,011	0,002	8,15E-10	0,020	0,015	0,162	-0,008	0,029	0,781	0,023	0,036	0,518	0,034	0,051	0,503
rs823114	1	Intergenic	A	G	0,009	0,002	2,31E-09	-0,024	0,012	0,050	0,018	0,025	0,469	-0,018	0,030	0,561	-0,029	0,043	0,506
rs11692435	2	Nonsynonymous:ACTR1B	A	G	0,017	0,003	2,53E-11	-0,019	0,023	0,406	0,129	0,044	0,004	0,006	0,057	0,914	0,219	0,075	0,005
rs1260326	2	Nonsynonymous:GCKR	C	T	0,021	0,002	8,05E-45	0,021	0,013	0,101	-0,021	0,025	0,398	-0,081	0,031	0,008	0,056	0,044	0,208
rs13024996	2	Intron:ARHGAP15	C	A	0,011	0,002	5,72E-13	0,030	0,013	0,018	0,053	0,026	0,037	0,016	0,031	0,617	0,054	0,045	0,227
rs13032049	2	Intron:WDPCP	G	A	0,010	0,002	3,00E-10	0,004	0,014	0,752	-0,017	0,027	0,541	-0,019	0,034	0,581	-0,039	0,049	0,425
rs13383034	2	Intron:LINC01833	T	C	0,015	0,002	6,31E-22	0,023	0,013	0,084	0,059	0,026	0,024	-0,008	0,033	0,799	-0,007	0,047	0,876
rs2178197	2	Intron:GPN1	A	G	0,009	0,002	2,45E-09	0,013	0,012	0,307	0,029	0,025	0,248	0,069	0,030	0,022	-0,039	0,044	0,375
rs56337305	2	Intergenic	T	C	0,010	0,002	1,63E-10	0,003	0,013	0,799	0,006	0,025	0,805	0,023	0,031	0,469	-0,008	0,045	0,860
rs72859280	2	Intergenic	T	G	0,023	0,004	4,44E-09	0,062	0,032	0,053	0,162	0,061	0,009	0,003	0,080	0,969	-0,018	0,117	0,878
rs77165542	2	Intergenic	C	T	0,026	0,004	5,63E-11	0,099	0,035	0,004	0,181	0,072	0,010	0,077	0,085	0,362	0,109	0,123	0,368
rs828867	2	Utr3:TET3	A	G	0,009	0,002	2,15E-09	0,008	0,013	0,546	0,014	0,025	0,568	0,036	0,031	0,246	-0,021	0,044	0,626
rs13066454	3	Intergenic	C	T	0,009	0,002	4,13E-09	-0,031	0,012	0,013	0,022	0,025	0,388	0,009	0,031	0,760	0,032	0,044	0,470
rs13094887	3	Intergenic	A	T	0,010	0,002	8,57E-11	0,005	0,013	0,689	-0,005	0,027	0,839	-0,020	0,033	0,553	0,036	0,047	0,449
rs2011092	3	Intron:ZBTB38	T	C	0,009	0,002	7,35E-09	0,010	0,013	0,425	0,007	0,026	0,795	-0,047	0,031	0,139	-0,016	0,045	0,730
rs60654199	3	Intron:RASA2	C	A	0,017	0,003	2,85E-08	-0,040	0,024	0,100	0,023	0,049	0,638	-0,042	0,059	0,481	0,021	0,087	0,808
rs62250685	3	Intron:CADM2	A	G	0,014	0,002	1,05E-21	0,013	0,013	0,309	0,018	0,025	0,463	0,017	0,031	0,572	-0,117	0,045	0,009
rs6787172	3	Intron:RSRC1	T	G	0,008	0,002	4,27E-08	-0,004	0,012	0,770	-0,003	0,025	0,916	0,034	0,030	0,258	0,027	0,043	0,536

rs74664784	3	Intron:CADM2	T	C	0,013	0,002	1,58E-14		0,195	0,352	0,568		0,303	0,771	0,680		-0,360	0,677	0,615		-0,186	1,076	0,867
rs9838144	3	Intron:CPNE4	G	C	0,010	0,002	2,65E-08		-0,018	0,015	0,226		-0,020	0,030	0,502		0,048	0,038	0,197		-0,027	0,053	0,613
rs10004020	4	Intergenic	A	G	0,009	0,002	2,43E-08		0,016	0,014	0,237		-0,007	0,027	0,799		-0,086	0,033	0,009		0,061	0,049	0,210
rs10028756	4	Intergenic	G	A	0,019	0,002	1,16E-17		0,042	0,018	0,020		0,004	0,036	0,916		0,085	0,046	0,060		0,178	0,068	0,007
rs11940694	4	Intron:KLB	G	A	0,026	0,002	3,03E-68		-0,010	0,013	0,420		0,022	0,025	0,381		0,002	0,031	0,946		-0,023	0,044	0,602
rs1229984	4	Nonsynonymous:ADH1B	C	T	0,151	0,004	<2E-308		-0,016	0,039	0,673		0,240	0,089	0,005		0,181	0,105	0,077		0,371	0,165	0,017
rs12499107	4	Intergenic	G	A	0,013	0,002	4,45E-09		0,007	0,018	0,691		-0,025	0,036	0,487		0,000	0,044	0,996		0,035	0,063	0,578
rs12651313	4	Intergenic	C	G	0,009	0,002	3,79E-09		0,012	0,012	0,346		0,002	0,025	0,936		0,023	0,030	0,442		-0,039	0,043	0,370
rs13107325	4	Nonsynonymous:SLC39A8	C	T	0,028	0,003	1,53E-22		-0,067	0,023	0,003		0,001	0,046	0,982		0,053	0,058	0,358		0,147	0,086	0,083
rs17029090	4	Synonymous:C4orf17	A	G	0,049	0,005	4,75E-21		-0,029	0,044	0,505		0,029	0,090	0,743		-0,157	0,101	0,129		-0,124	0,145	0,404
rs2165670	4	Intergenic	A	G	0,023	0,002	1,67E-22		0,025	0,020	0,207		0,070	0,039	0,079		0,094	0,048	0,053		0,003	0,072	0,972
rs36052336	4	Intron:ADH1C	A	G	0,018	0,003	1,23E-09		-0,017	0,025	0,510		-0,011	0,050	0,828		-0,079	0,060	0,190		-0,030	0,088	0,736
rs3748034	4	Nonsynonymous:HGFAC	G	T	0,012	0,002	1,67E-08		0,016	0,018	0,377		-0,044	0,035	0,208		-0,018	0,043	0,681		-0,101	0,060	0,093
rs4501255	4	Intron:BEND4	G	C	0,011	0,002	4,83E-10		-0,014	0,014	0,339		0,056	0,028	0,047		0,015	0,035	0,680		-0,020	0,051	0,695
rs4690727	4	Intron:INPP4B	G	C	0,011	0,002	2,43E-11		-0,011	0,014	0,424		-0,016	0,027	0,552		-0,015	0,034	0,647		0,005	0,048	0,924
rs4699791	4	Intergenic	A	G	0,019	0,003	6,58E-14		-0,006	0,021	0,786		0,034	0,041	0,399		0,025	0,050	0,624		0,008	0,073	0,910
rs561222871	4	Intron:ADH1C	C	T	0,039	0,004	6,56E-27		-0,065	0,422	0,878		0,572	1,080	0,572		-0,890	0,797	0,314		-0,421	1,404	0,776
rs7682824	4	Intergenic	T	C	0,008	0,002	2,77E-08		-0,014	0,017	0,409		-0,022	0,035	0,523		-0,016	0,043	0,705		0,101	0,060	0,094
rs79139602	4	Intron:C4orf17	T	A	0,060	0,005	1,80E-32		0,018	0,043	0,677		-0,044	0,089	0,619		0,147	0,100	0,150		0,099	0,145	0,500
rs11739827	5	Intron:TENM2	G	T	0,008	0,002	1,18E-08		0,000	0,012	0,987		0,007	0,025	0,793		-0,026	0,031	0,395		0,018	0,044	0,683
rs12655091	5	Intergenic	G	A	0,008	0,002	1,25E-08		-0,009	0,012	0,460		-0,002	0,025	0,945		0,026	0,030	0,394		-0,068	0,043	0,116
rs4916723	5	Intron:LINC00461	A	C	0,010	0,002	1,72E-11		0,004	0,013	0,724		0,006	0,025	0,800		-0,042	0,031	0,176		0,034	0,044	0,447
rs55872084	5	Intron:SGCD	T	G	0,010	0,002	6,32E-09		-0,013	0,014	0,373		-0,026	0,029	0,368		-0,014	0,035	0,684		-0,017	0,050	0,738
rs10085696	7	Intron:AUTS2	A	G	0,011	0,002	1,12E-09		-0,026	0,016	0,098		0,048	0,032	0,133		-0,007	0,039	0,867		0,026	0,056	0,645
rs10236149	7	Intron:ARPC1B	A	G	0,014	0,002	1,18E-09		-0,010	0,018	0,582		0,006	0,037	0,870		0,027	0,046	0,550		0,144	0,069	0,032
rs35034355	7	Intron:ORC5	G	A	0,008	0,002	2,87E-08		0,001	0,012	0,930		0,008	0,024	0,729		-0,010	0,030	0,730		-0,019	0,043	0,663
rs6460047	7	Intergenic	C	T	0,012	0,002	9,69E-11		-0,001	0,015	0,939		0,031	0,030	0,298		0,039	0,037	0,295		-0,059	0,054	0,271

rs6951574	7	Intergenic	C	T	0,013	0,002	1,58E-19		-0,012	0,012	0,336		-0,004	0,025	0,863		-0,012	0,030	0,690		0,046	0,044	0,287
rs1217091	8	Intergenic	C	T	0,012	0,002	7,05E-11		0,022	0,016	0,159		0,080	0,032	0,012		0,033	0,039	0,390		0,068	0,056	0,221
rs13250583	8	Intergenic	C	T	0,010	0,002	4,70E-08		0,003	0,015	0,831		-0,030	0,030	0,313		0,018	0,037	0,620		0,008	0,053	0,882
rs28601761	8	Intergenic	G	C	0,009	0,002	7,17E-10		0,020	0,013	0,106		-0,006	0,025	0,817		-0,066	0,031	0,033		-0,101	0,045	0,023
rs10978550	9	Intergenic	T	C	0,012	0,002	7,15E-11		0,009	0,015	0,554		0,021	0,031	0,490		0,039	0,038	0,306		0,037	0,054	0,493
rs55932213	9	Intergenic	G	A	0,010	0,002	9,55E-09		-0,002	0,014	0,867		-0,063	0,028	0,026		0,001	0,035	0,988		0,083	0,052	0,104
rs17665139	10	Intergenic	C	T	0,012	0,002	1,59E-08		-0,013	0,017	0,452		0,006	0,034	0,869		-0,012	0,042	0,781		0,036	0,061	0,557
rs7074871	10	Intergenic	G	A	0,009	0,002	1,86E-08		-0,005	0,014	0,743		0,019	0,028	0,491		0,032	0,035	0,354		-0,058	0,049	0,231
rs10750025	11	Intergenic	T	C	0,010	0,002	4,89E-11		-0,005	0,013	0,699		0,014	0,026	0,599		-0,015	0,032	0,652		0,051	0,047	0,271
rs11030084	11	Intron:BDNF-AS|LINC00678	C	T	0,011	0,002	1,72E-08		-0,009	0,016	0,567		0,044	0,032	0,165		0,037	0,039	0,339		0,032	0,056	0,570
rs12795042	11	Intron:LOC646522	A	C	0,008	0,002	3,25E-08		-0,018	0,013	0,165		-0,019	0,026	0,468		0,018	0,031	0,558		0,031	0,045	0,485
rs1713676	11	Intergenic	A	G	0,008	0,002	4,29E-08		-0,007	0,012	0,568		0,013	0,024	0,602		0,035	0,030	0,250		0,021	0,043	0,628
rs4938230	11	Intergenic	A	C	0,013	0,002	1,48E-10		0,029	0,017	0,090		0,017	0,034	0,612		-0,037	0,042	0,379		0,061	0,062	0,318
rs56030824	11	Intron:SPI1	G	A	0,012	0,002	1,15E-13		0,016	0,013	0,214		0,016	0,026	0,532		0,008	0,032	0,804		0,058	0,047	0,210
rs682011	11	Intergenic	C	T	0,008	0,002	2,22E-08		-0,002	0,012	0,874		0,008	0,025	0,736		-0,012	0,030	0,688		-0,013	0,043	0,759
rs7950166	11	Intron:TRIM66	C	T	0,010	0,002	9,89E-11		-0,010	0,013	0,456		0,024	0,025	0,337		-0,078	0,032	0,013		-0,034	0,045	0,451
rs10506274	12	Intron:ACSS3	G	T	0,009	0,002	5,78E-10		0,018	0,012	0,133		0,046	0,024	0,059		-0,037	0,030	0,220		-0,072	0,043	0,094
rs10876188	12	Intron:SLC4A8	C	T	0,008	0,002	4,84E-08		-0,014	0,012	0,249		-0,009	0,025	0,702		0,026	0,030	0,396		-0,006	0,043	0,894
rs3809162	12	Intergenic	G	A	0,009	0,002	1,19E-09		-0,014	0,012	0,263		0,017	0,025	0,496		-0,052	0,031	0,093		-0,034	0,044	0,445
rs4842786	12	Intergenic	G	A	0,009	0,002	2,73E-09		-0,007	0,012	0,564		-0,019	0,025	0,453		-0,018	0,031	0,552		-0,011	0,044	0,800
rs500321	13	Intergenic	A	T	0,010	0,002	4,92E-09		-0,012	0,014	0,366		-0,007	0,028	0,800		-0,004	0,034	0,907		-0,025	0,049	0,602
rs1123285	14	Intron:OTX2	C	G	0,009	0,002	8,14E-09		0,003	0,013	0,834		-0,003	0,026	0,902		-0,040	0,032	0,213		0,058	0,047	0,212
rs11625650	14	Intron:KIF26A	G	A	0,010	0,002	2,89E-08		-0,014	0,014	0,334		0,042	0,029	0,145		0,006	0,035	0,875		-0,022	0,050	0,656
rs2180870	14	Intron:ARID4A	T	C	0,012	0,002	1,12E-08		0,029	0,018	0,116		0,030	0,037	0,410		-0,059	0,044	0,183		0,017	0,064	0,796
rs28929474	14	Nonsynonymous:SERPINA1	C	T	0,037	0,005	1,34E-11		-0,172	0,040	0,000		0,151	0,094	0,100		0,185	0,116	0,103		-0,189	0,139	0,186
rs12907323	15	Intron:AGBL1	G	A	0,009	0,002	9,93E-09		-0,007	0,013	0,555		0,013	0,025	0,593		-0,020	0,031	0,521		0,031	0,044	0,475
rs2472297	15	Intergenic	T	C	0,011	0,002	3,1E-10		0,003	0,014	0,834		0,026	0,028	0,340		0,015	0,034	0,657		0,019	0,049	0,704

rs1104608	16	Intergenic	G	C	0,011	0,002	1,05E-13		0,004	0,012	0,768		0,058	0,025	0,021		0,005	0,031	0,874		-0,012	0,044	0,785
rs113443718	16	Intron:SEZ6L2	G	A	0,010	0,002	1,19E-10		-0,010	0,013	0,449		-0,007	0,026	0,788		-0,043	0,032	0,181		0,028	0,047	0,548
rs17177078	16	Intron:TNRC6A	C	T	0,022	0,003	1,27E-13		-0,015	0,026	0,564		0,130	0,057	0,019		-0,002	0,066	0,974		0,066	0,097	0,490
rs2764771	16	Intergenic	A	G	0,010	0,002	4,02E-10		-0,008	0,013	0,552		-0,080	0,027	0,003		-0,037	0,033	0,257		-0,036	0,047	0,448
rs378421	16	Intergenic	G	A	0,011	0,002	4,83E-14		-0,003	0,012	0,792		-0,002	0,025	0,952		-0,001	0,031	0,982		-0,046	0,044	0,289
rs62044525	16	Intergenic	C	G	0,012	0,002	1,03E-10		0,007	0,016	0,661		0,018	0,031	0,571		0,078	0,039	0,047		-0,020	0,055	0,712
rs7185555	16	Intergenic	G	C	0,011	0,002	4,24E-08		-0,003	0,017	0,879		-0,016	0,033	0,622		0,015	0,041	0,716		-0,004	0,059	0,940
rs79616692	16	Intron:LINC01572	C	G	0,016	0,002	4,11E-12		-0,010	0,020	0,614		-0,003	0,040	0,933		0,001	0,049	0,980		0,005	0,070	0,943
rs10438820	17	Intron:RPTOR	T	C	0,009	0,002	1,76E-08		0,007	0,013	0,616		-0,012	0,027	0,658		0,024	0,033	0,465		0,021	0,047	0,655
rs2854334	17	Intergenic	G	A	0,009	0,002	7,51E-10		-0,008	0,013	0,538		-0,001	0,025	0,979		-0,031	0,031	0,311		0,028	0,045	0,524
rs3803800	17	Nonsynonymous:TNFSF 12-TNFSF13|TNFSF13	G	A	0,011	0,002	1,5E-10		0,009	0,015	0,566		-0,013	0,030	0,675		-0,070	0,036	0,054		0,044	0,053	0,413
rs4548913	17	Intron:SRR	G	A	0,008	0,002	3,11E-08		0,014	0,013	0,271		0,023	0,025	0,374		-0,036	0,032	0,256		0,057	0,045	0,201
rs4092465	18	Intergenic	A	G	0,008	0,002	4,39E-08		-0,015	0,013	0,256		0,013	0,026	0,624		-0,070	0,032	0,029		0,014	0,045	0,760
rs9950000	18	Intron:TCF4	C	T	0,009	0,002	9,38E-10		-0,026	0,013	0,042		-0,008	0,025	0,753		0,012	0,031	0,707		0,004	0,044	0,930
rs281379	19	Intergenic	A	G	0,014	0,002	4,91E-21		0,053	0,012	0,000		0,038	0,025	0,124		0,032	0,030	0,291		0,032	0,043	0,467
rs4815364	20	Intron:ACSS1	A	G	0,009	0,002	1,02E-08		0,033	0,013	0,008		0,009	0,025	0,724		0,010	0,031	0,749		0,008	0,044	0,851
rs9607814	22	Intergenic	C	A	0,010	0,002	4,31E-08		-0,013	0,015	0,392		0,020	0,031	0,513		-0,032	0,038	0,400		0,051	0,056	0,358

*Data are from genome-wide association studies on alcohol consumption[1] and from UK Biobank. †The betas for alcohol consumption are per SD increase of log-transformed drinks per week and the betas for the cardiovascular diseases are log odds ratios.

Table VIII. Associations of alcohol consumption instrumented by 94 SNPs with blood pressure and serum lipids in UK Biobank

Outcome	Unit	Method	Beta	LB	UB	P value	SNPs	Egger, P value	No. of outliers
Systolic blood pressure	mm Hg	Univariable IVW	0.124	0.051	0.198	0.001	94		
Systolic blood pressure	mm Hg	Weighted median	0.171	0.095	0..248	<0.001	94		
Systolic blood pressure	mm Hg	MR-Egger	0.259	0.122	0.395	<0.001	94	0.024	
Systolic blood pressure	mm Hg	MR-PRESSO	0.072	0.003	0.140	0.043	94		10
Systolic blood pressure	mm Hg	MVMR (smoking-adjusted)	0.150	0.066	0.234	<0.001	94		
Diastolic blood pressure	mm Hg	Univariable IVW	0.152	0.053	0.249	0.003	94		
Diastolic blood pressure	mm Hg	Weighted median	0.141	0.057	0.224	0.001	94		
Diastolic blood pressure	mm Hg	MR-Egger	0.220	0.032	0.408	0.022	94	0.401	
Diastolic blood pressure	mm Hg	MR-PRESSO	0.123	0.045	0.201	0.003	94		11
Diastolic blood pressure	mm Hg	MVMR (smoking-adjusted)	0.131	0.018	0.244	0.023	94		
HDL cholesterol	mmol/L	Univariable IVW	0.386	0.246	0.525	<0.001	94		
HDL cholesterol	mmol/L	Weighted median	0.220	0.129	0.312	<0.001	94		
HDL cholesterol	mmol/L	MR-Egger	0.079	-0.179	0.337	0.547	94	0.006	
HDL cholesterol	mmol/L	MR-PRESSO	0.372	0.301	0.444	<0.001	94		14
HDL cholesterol	mmol/L	MVMR (smoking-adjusted)	0.351	0.191	0.511	<0.001	94		
LDL cholesterol	mmol/L	Univariable IVW	-0.011	-0.018	0.159	0.900	94		
LDL cholesterol	mmol/L	Weighted median	0.030	-0.057	0.116	0.500	94		
LDL cholesterol	mmol/L	MR-Egger	0.275	-0.045	0.595	0.092	94	0.040	
LDL cholesterol	mmol/L	MR-PRESSO	-0.046	-0.114	0.023	0.195	94		10
LDL cholesterol	mmol/L	MVMR (smoking-adjusted)	0.033	-0.160	0.226	0.740	94		
Triglycerides	log-mmol/L	Univariable IVW	-0.371	-0.067	-0.069	0.016	94		
Triglycerides	log-mmol/L	Weighted median	-0.088	-0.179	0.004	0.060	94		
Triglycerides	log-mmol/L	MR-Egger	-0.303	-0.886	0.280	0.309	94	0.787	
Triglycerides	log-mmol/L	MR-PRESSO	-0.0212	-0.292	-0.133	<0.001	94		21
Triglycerides	log-mmol/L	MVMR (smoking-adjusted)	-0.456	-0.807	-0.104	0.011	94		

*IVW, inverse-variance weighted; LB, lower bound of the 95% confidence interval for the beta; MR-PRESSO, Mendelian randomization Pleiotropy RESidual Sum and Outlier; MVMR, multivariable Mendelian randomization; UB, upper bound of the 95% confidence interval of the beta.

Table IX. Associations of alcohol consumption instrumented by rs1229984 in *ADH1B* with blood pressure and serum lipids in UK Biobank

Outcome	Unit	Beta	LB	UB	P Value
Systolic blood pressure	mmHg	0.305	0.231	0.378	3.61e-16
Diastolic blood pressure	mmHg	0.125	0.030	0.221	0.010
HDL cholesterol	mmol/L	0.000	-0.094	0.095	0.992
LDL cholesterol	mmol/L	0.397	0.298	0.495	4.01e-15
Triglycerides	log-mmol/L	-0.084	-0.180	0.013	0.089

*LB, lower bound of the 95% confidence interval for the beta; UB, upper bound of the 95% confidence interval of the beta.

Table X. Associations of alcohol consumption instrumented by up to 94 SNPs with cardiovascular disease using data from consortia and UK Biobank

Outcome	Data source	Method	OR	LB	UB	P value	SNPs	Egger, P value	No. of outliers
Stroke	MEGASTROKE/UK Biobank	Univariable IVW	1.270	1.116	1.445	2.87e-4	92/94*		
Stroke	MEGASTROKE/UK Biobank	Weighted median	1.298	1.070	1.575	0.008	92/94*		
Stroke	MEGASTROKE/UK Biobank	MR-Egger	1.205	0.974	1.490	0.085	92/94*	0.56/0.90*	
Stroke	MEGASTROKE/UK Biobank	MR-PRESSO	1.270	1.116	1.445	2.87e-4	92/94*		0/0*
Stroke	MEGASTROKE/UK Biobank	MVMR (smoking-adjusted)	1.210	1.046	1.400	0.010	92/94*		
Coronary artery disease	CARDIoGRAMplusC4D/UK Biobank	Univariable IVW	1.164	0.998	1.358	0.052	93/94*		
Coronary artery disease	CARDIoGRAMplusC4D/UK Biobank	Weighted median	1.309	1.091	1.571	0.004	93/94*		
Coronary artery disease	CARDIoGRAMplusC4D/UK Biobank	MR-Egger	1.470	1.146	1.885	0.002	93/94*	0.11/0.05*	
Coronary artery disease	CARDIoGRAMplusC4D/UK Biobank	MR-PRESSO	1.162	1.019	1.326	0.026	93/94*		3/5*
Coronary artery disease	CARDIoGRAMplusC4D/UK Biobank	MVMR (smoking-adjusted)	1.077	0.910	1.275	0.389	93/94*		
Atrial fibrillation	Atrial Fibrillation Consortium	Univariable IVW	1.170	1.000	1.369	0.050	94		
Atrial fibrillation	Atrial Fibrillation Consortium	Weighted median	1.166	0.929	1.463	0.186	94		
Atrial fibrillation	Atrial Fibrillation Consortium	MR-Egger	1.238	0.944	1.624	0.124	94	0.62	
Atrial fibrillation	Atrial Fibrillation Consortium	MR-PRESSO	1.202	1.043	1.385	0.013	94		1
Atrial fibrillation	Atrial Fibrillation Consortium	MVMR (smoking-adjusted)	1.112	0.933	1.323	0.237	94		
Heart failure	UK Biobank	Univariable IVW	1.001	0.682	1.469	0.996	94		
Heart failure	UK Biobank	Weighted median	1.605	0.902	2.858	0.108	94		
Heart failure	UK Biobank	MR-Egger	1.347	0.636	2.851	0.436	94	0.37	
Heart failure	UK Biobank	MR-PRESSO	1.001	0.682	1.469	0.996	94		0
Heart failure	UK Biobank	MVMR (smoking-adjusted)	0.880	0.566	1.368	0.570	94		
Venous thromboembolism	UK Biobank	Univariable IVW	1.036	0.774	1.387	0.810	94		
Venous thromboembolism	UK Biobank	Weighted median	0.897	0.585	1.375	0.619	94		
Venous thromboembolism	UK Biobank	MR-Egger	0.958	0.547	1.676	0.879	94	0.75	
Venous thromboembolism	UK Biobank	MR-PRESSO	1.026	0.790	1.332	0.849	94		2
Venous thromboembolism	UK Biobank	MVMR (smoking-adjusted)	1.048	0.748	1.468	0.784	94		
Peripheral artery disease	UK Biobank	Univariable IVW	3.051	1.921	4.846	2.30e-6	94		

Peripheral artery disease	UK Biobank	Weighted median	3.796	1.777	8.110	0.001	94		
Peripheral artery disease	UK Biobank	MR-Egger	5.434	2.137	13.81	3.80e-4	94	0.16	
Peripheral artery disease	UK Biobank	MR-PRESSO	3.051	1.921	4.846	2.30e-6	94		0
Peripheral artery disease	UK Biobank	MVMR (smoking-adjusted)	2.550	1.493	4.354	6.09e-4	94		
Aortic valve stenosis	UK Biobank	Univariable IVW	1.029	0.558	1.898	0.926	94		
Aortic valve stenosis	UK Biobank	Weighted median	2.744	1.096	6.873	0.031	94		
Aortic valve stenosis	UK Biobank	MR-Egger	3.347	1.021	10.96	0.046	94	0.02	
Aortic valve stenosis	UK Biobank	MR-PRESSO	1.029	0.558	1.898	0.926	94		0
Aortic valve stenosis	UK Biobank	MVMR (smoking-adjusted)	0.939	0.462	1.910	0.862	94		
Abdominal aortic aneurysm	UK Biobank	Univariable IVW	2.602	1.150	5.891	0.022	94		
Abdominal aortic aneurysm	UK Biobank	Weighted median	9.281	2.423	35.56	0.001	94		
Abdominal aortic aneurysm	UK Biobank	MR-Egger	7.890	1.477	42.13	0.016	94	0.14	
Abdominal aortic aneurysm	UK Biobank	MR-PRESSO	2.602	1.150	5.891	0.022	94		0
Abdominal aortic aneurysm	UK Biobank	MVMR (smoking-adjusted)	2.293	0.886	5.930	0.087	94		

*Consortium/UK Biobank. The consortium is MEGASTROKE for stroke and CARDIoGRAMplusC4D for coronary artery disease. CI, confidence interval; IVW, inverse-variance weighted; LB, lower bound of the 95% CI; MR-PRESSO, Mendelian randomization Pleiotropy RESidual Sum and Outlier; MVMR, multivariable Mendelian randomization; OR, odds ratio; UB, upper bound of the 95% CI.

Table XI. Associations of alcohol consumption instrumented by 94 SNPs with cardiovascular diseases in 197 822 never smokers in UK Biobank*

Outcome	Cases	OR	95% CI	P Value
Stroke	3980	1.26	0.83-1.92	0.282
Coronary artery disease	11 221	1.39	0.98-1.96	0.064
Atrial fibrillation	7379	1.21	0.84-175	0.301
Heart failure	2422	1.13	0.62-2.03	0.692
Venous thromboembolism	6760	1.10	0.74-1.63	0.652
Peripheral artery disease	854	1.75	0.72-4.29	0.220
Aortic valve stenosis	847	1.15	0.44-2.99	0.775
Abdominal aortic aneurysm	167	NA†	NA†	NA†

*Analyses were conducted using the random-effects inverse variance weighted method.
†Not applicable; too few cases for analysis.

Table XII. Associations of alcohol consumption instrumented by 94 SNPs with stroke and atrial fibrillation in European-descent individuals in the MEGASTROKE and AFGen

Outcome	Data source	OR	LB	UB	P value
Any stroke	MEGASTROKE	1.19	0.99	1.43	0.061
Any ischemic stroke	MEGASTROKE	1.20	0.98	1.48	0.077
Large artery stroke	MEGASTROKE	1.24	0.77	2.02	0.380
Small vessel stroke	MEGASTROKE	1.24	0.77	2.01	0.376
Cardioembolic stroke	MEGASTROKE	1.09	0.74	1.61	0.671
Atrial fibrillation	Atrial Fibrillation Consortium	1.13	0.95	1.34	0.165

*LB, lower bound of the 95% confidence interval for the OR; OR, odds ratio; UB, upper bound of the 95% confidence interval of the OR.

Table XIII. Associations of alcohol intake instrumented by rs1229984 in *ADH1B* with cardiovascular disease

Outcome	Data source	OR	LB	UB	P Value
Stroke	MEGASTROKE and UK Biobank	1.33	1.06	1.62	0.014
Coronary artery disease	CARDIoGRAMplusC4D and UK Biobank	1.48	1.21	1.82	1.91E-4
Atrial fibrillation	Atrial Fibrillation Consortium	1.17	0.91	1.49	0.216
Heart failure	UK Biobank	1.71	0.80	3.66	0.163
Venous thromboembolism	UK Biobank	0.90	0.54	1.49	0.673
Peripheral artery disease	UK Biobank	4.91	1.55	15.57	0.005
Aortic valve stenosis	UK Biobank	3.32	0.85	13.04	0.077
Abdominal aortic aneurysm	UK Biobank	11.79	1.37	101.18	0.017

*LB, lower bound of the 95% confidence interval for the OR; OR, odds ratio; SNPs, single-nucleotide polymorphism; UB, upper bound of the 95% confidence interval of the OR.

Table XIV. Associations of genetically predicted alcohol consumption instrumented by up to 93 SNPs, excluding rs1229984 in *ADH1B*, with cardiovascular disease

Outcome	Data source	Method	OR	LB	UB	P Value	SNPs
Stroke	MEGASTROKE/UK Biobank	Univariable IVW	1.257	1.078	1.467	0.004	91/93*
Stroke	MEGASTROKE/UK Biobank	Weighted median	1.088	0.870	1.360	0.462	91/93*
Stroke	MEGASTROKE/UK Biobank	MR-Egger	1.033	0.714	1.494	0.863	91/93*
Stroke	MEGASTROKE/UK Biobank	MR-PRESSO	1.257	1.078	1.467	0.004	91/93*
Stroke	MEGASTROKE/UK Biobank	MVMR (smoking-adjusted)	1.169	1.020	1.340	0.025	91/93*
Coronary artery disease	CARDIoGRAMplusC4D/UK Biobank	Univariable IVW	1.073	0.892	1.290	0.453	92/93*
Coronary artery disease	CARDIoGRAMplusC4D/UK Biobank	Weighted median	1.255	1.012	1.557	0.039	92/93*
Coronary artery disease	CARDIoGRAMplusC4D/UK Biobank	MR-Egger	1.564	0.982	2.494	0.060	92/93*
Coronary artery disease	CARDIoGRAMplusC4D/UK Biobank	MR-PRESSO	1.148	1.005	1.312	0.042	92/93*
Coronary artery disease	CARDIoGRAMplusC4D/UK Biobank	MVMR (smoking-adjusted)	0.955	0.825	1.105	0.536	92/93*
Atrial fibrillation	Atrial Fibrillation Consortium	Univariable IVW	1.171	0.976	1.404	0.090	93
Atrial fibrillation	Atrial Fibrillation Consortium	Weighted median	1.060	0.843	1.334	0.619	93
Atrial fibrillation	Atrial Fibrillation Consortium	MR-Egger	1.366	0.869	2.146	0.176	93
Atrial fibrillation	Atrial Fibrillation Consortium	MR-PRESSO	1.213	1.029	1.431	0.024	93
Atrial fibrillation	Atrial Fibrillation Consortium	MVMR (smoking-adjusted)	1.088	0.878	1.347	0.442	93
Heart failure	UK Biobank	Univariable IVW	0.888	0.582	1.355	0.581	93
Heart failure	UK Biobank	Weighted median	0.895	0.508	1.579	0.703	93
Heart failure	UK Biobank	MR-Egger	0.870	0.265	2.859	0.819	93
Heart failure	UK Biobank	MR-PRESSO	1.439	0.991	2.091	0.059	93
Heart failure	UK Biobank	MVMR (smoking-adjusted)	1.231	0.791	1.917	0.357	93
Venous thromboembolism	UK Biobank	Univariable IVW	1.074	0.775	1.488	0.670	93
Venous thromboembolism	UK Biobank	Weighted median	0.944	0.634	1.404	0.776	93
Venous thromboembolism	UK Biobank	MR-Egger	1.089	0.439	2.700	0.855	93
Venous thromboembolism	UK Biobank	MR-PRESSO	1.061	0.791	1.424	0.692	93

Venous thromboembolism	UK Biobank	MVMR (smoking-adjusted)	0.958	0.734	1.251	0.754	93
Peripheral artery disease	UK Biobank	Univariable IVW	2.790	1.683	4.622	6.87E-5	93
Peripheral artery disease	UK Biobank	Weighted median	2.279	1.041	4.986	0.039	93
Peripheral artery disease	UK Biobank	MR-Egger	5.779	1.406	23.75	0.015	93
Peripheral artery disease	UK Biobank	MR-PRESSO	2.790	1.683	4.622	6.87E-5	93
Peripheral artery disease	UK Biobank	MVMR (smoking-adjusted)	1.522	0.963	2.408	0.073	93
Aortic valve stenosis	UK Biobank	Univariable IVW	0.812	0.418	1.578	0.540	93
Aortic valve stenosis	UK Biobank	Weighted median	1.081	0.378	3.094	0.884	93
Aortic valve stenosis	UK Biobank	MR-Egger	2.863	0.467	17.55	0.255	93
Aortic valve stenosis	UK Biobank	MR-PRESSO	0.812	0.418	1.578	0.540	93
Aortic valve stenosis	UK Biobank	MVMR (smoking-adjusted)	0.704	0.402	1.235	0.221	93
Abdominal aortic aneurysm	UK Biobank	Univariable IVW	2.016	0.833	4.878	0.120	93
Abdominal aortic aneurysm	UK Biobank	Weighted median	2.408	0.623	9.310	0.203	93
Abdominal aortic aneurysm	UK Biobank	MR-Egger	4.333	0.385	48.81	0.235	93
Abdominal aortic aneurysm	UK Biobank	MR-PRESSO	2.016	0.833	4.878	0.120	93
Abdominal aortic aneurysm	UK Biobank	MVMR (smoking-adjusted)	1.102	0.496	2.447	0.812	93

*Consortium/UK Biobank. The consortium is MEGASTROKE for stroke and CARDIoGRAMplusC4D for coronary artery disease. ADH1B, alcohol dehydrogenase 1B; CVD, cardiovascular disease; CI, confidence interval; IVW, inverse-variance weighted; LB, lower bound of the 95% CI; MR-PRESSO, Mendelian randomization Pleiotropy RESidual Sum and Outlier; MVMR multivariable Mendelian randomization; OR, odds ratio; UB, upper bound of the 95% CI; SNP, single-nucleotide polymorphism.

REFERENCES

1. Liu M, Jiang Y, Wedow R, Li Y, Brazel DM, Chen F, Datta G, Davila-Velderrain J, McGuire D, Tian C, et al. Association studies of up to 1.2 million individuals yield new insights into the genetic etiology of tobacco and alcohol use. *Nat Genet.* 2019;51:237-244.
2. Lee JJ, Wedow R, Okbay A, Kong E, Maghzian O, Zacher M, Nguyen-Viet TA, Bowers P, Sidorenko J, Karlsson Linner R, et al. Gene discovery and polygenic prediction from a genome-wide association study of educational attainment in 1.1 million individuals. *Nat Genet.* 2018;50:1112-1121.
3. Sudlow C, Gallacher J, Allen N, Beral V, Burton P, Danesh J, Downey P, Elliott P, Green J, Landray M, et al. UK biobank: an open access resource for identifying the causes of a wide range of complex diseases of middle and old age. *PLoS Med.* 2015;12:e1001779.
4. Malik R, Chauhan G, Traylor M, Sargurupremraj M, Okada Y, Mishra A, Rutten-Jacobs L, Giese AK, van der Laan SW, Gretarsdottir S, et al. Multiancestry genome-wide association study of 520,000 subjects identifies 32 loci associated with stroke and stroke subtypes. *Nat Genet.* 2018 50:524-537.
5. Woo D, Falcone GJ, Devan WJ, Brown WM, Biffi A, Howard TD, Anderson CD, Brouwers HB, Valant V, Battey TW, et al. Meta-analysis of genome-wide association studies identifies 1q22 as a susceptibility locus for intracerebral hemorrhage. *Am J Hum Genet.* 2014;94:511-521.
6. Nikpay M, Goel A, Won HH, Hall LM, Willenborg C, Kanoni S, Saleheen D, Kyriakou T, Nelson CP, Hopewell JC, et al. A comprehensive 1,000 Genomes-based genome-wide association meta-analysis of coronary artery disease. *Nat Genet.* 2015;47:1121-1130.
7. Roselli C, Chaffin MD, Weng LC, Aeschbacher S, Ahlberg G, Albert CM, Almgren P, Alonso A, Anderson CD, Aragam KG, et al. Multi-ethnic genome-wide association study for atrial fibrillation. *Nat Genet.* 2018;50:1225-1233.
8. Machiela MJ, Chanock SJ. LDlink: a web-based application for exploring population-specific haplotype structure and linking correlated alleles of possible functional variants. *Bioinformatics.* 2015;31:3555-3557.
9. Burgess S, Bowden J, Fall T, Ingelsson E, Thompson SG. Sensitivity analyses for robust causal inference from Mendelian randomization analyses with multiple genetic variants. *Epidemiology.* 2017;28:30-42.
10. Verbanck M, Chen CY, Neale B, Do R. Detection of widespread horizontal pleiotropy in causal relationships inferred from Mendelian randomization between complex traits and diseases. *Nat Genet.* 2018;50:693-698.
11. Burgess S, Thompson SG. Multivariable Mendelian randomization: the use of pleiotropic genetic variants to estimate causal effects. *Am J Epidemiol.* 2015;181:251-260.
12. Sanderson E, Davey Smith G, Windmeijer F, Bowden J. An examination of multivariable Mendelian randomization in the single-sample and two-sample summary data settings. *Int J Epidemiol.* 2019;48:713-727.
13. Spiller W, Davies NM, Palmer TM. Software application profile: mrrobust—a tool for performing two-sample summary Mendelian randomization analyses *Int J Epidemiol.* 2019;48:684–690.
14. Yavorska OO, Burgess S. MendelianRandomization: an R package for performing Mendelian randomization analyses using summarized data. *Int J Epidemiol.* 2017;46:1734-1739.
15. Brion MJ, Shakhbazov K, Visscher PM. Calculating statistical power in Mendelian randomization studies. *Int J Epidemiol.* 2013;42:1497-1501.
16. Nikpay M, Goel A, Won HH, Hall LM, Willenborg C, Kanoni S, Saleheen D, Kyriakou T, Nelson CP, Hopewell JC, et al. A comprehensive 1,000 Genomes-based genome-wide association meta-analysis of coronary artery disease. *Nat Genet.* 2015;47:1121-1130.
